# Joint Mixture Associations of Prenatal Air Pollution and Polyunsaturated Fatty Acid Intake on Birth Weight Outcomes in an African American Cohort

**DOI:** 10.1021/envhealth.5c00490

**Published:** 2026-04-22

**Authors:** Benjamin R. Kessler, Anne L. Dunlop, Robert B. Hood, Stephanie M. Eick, Jiada James Zhan, Susan S. Hoffman, Zhihao Jin, Youran Tan, Erin Ferranti, Jeremy Sarnat, Audrey J. Gaskins, Howard H. Chang, Christine Ekenga, Donghai Liang

**Affiliations:** † Gangaros Department of Environmental Health, 1371 Emory University, Atlanta, Georgia 30322, United States; ‡ School of Medicine Department of Gynecology & Obstetrics, Emory University, Atlanta, Georgia 30322, United States; § College of Public Health, The Ohio State University, Columbus, Ohio 43210, United States; ∥ Nutrition & Health Sciences Doctoral Program, 25798 Emory University, Atlanta, Georgia 30322, United States; ⊥ Department of Epidemiology, Emory University, Atlanta, Georgia 30322, United States; # Department of Gynecology & Obstetrics, Nell Hodgson Woodruff School of Nursing, Emory University, Atlanta, Georgia 30322, United States; 7 Department of Biostatistics & Bioinformatics, Emory University, Atlanta, Georgia 30322, United States

**Keywords:** maternal health, diet, health disparities, omega-3, omega-6

## Abstract

Dietary polyunsaturated fatty acids (PUFAs) play a critical role in healthy pregnancy by supporting fetal development and modulating inflammation. Few studies have considered how dietary PUFAs and environmental exposures may jointly influence birth outcomes. We therefore investigated the joint mixture associations of prenatal exposure to traffic-related air pollutants (TRAPs), including carbon monoxide, fine particulate matter (PM_2.5_), nitrogen oxides, and dietary PUFA intake, on birth weight for gestational age z-score (BWZ) in 297 pregnant African Americans enrolled in the Atlanta Maternal-Child Cohort. At mean α-linolenic acid (ALA) intake, a first-trimester interquartile range (IQR) increase in PM_2.5_ was associated with a 0.08 (95% CI: 0.02–0.18) unit decrease in BWZ, but at twice the mean ALA intake, the same PM_2.5_ increase was associated with a 1.12 (95% CI: 0.19–2.05) unit increase in BWZ (*p*-int = 0.04). Similarly, at mean linoleic acid (LA) intake, a first-trimester IQR increase in PM_2.5_ was associated with a 0.08 (95% CI: 0.02–0.17) unit decrease in BWZ, whereas at twice the mean LA intake, PM_2.5_ exposure was associated with a 1.09 (95% CI, 0.16–2.02) unit increase (*p*-int = 0.09). Our findings suggest that higher prenatal PUFA intake may mitigate the adverse effects of air pollution on fetal growth.

## Graphical abstract

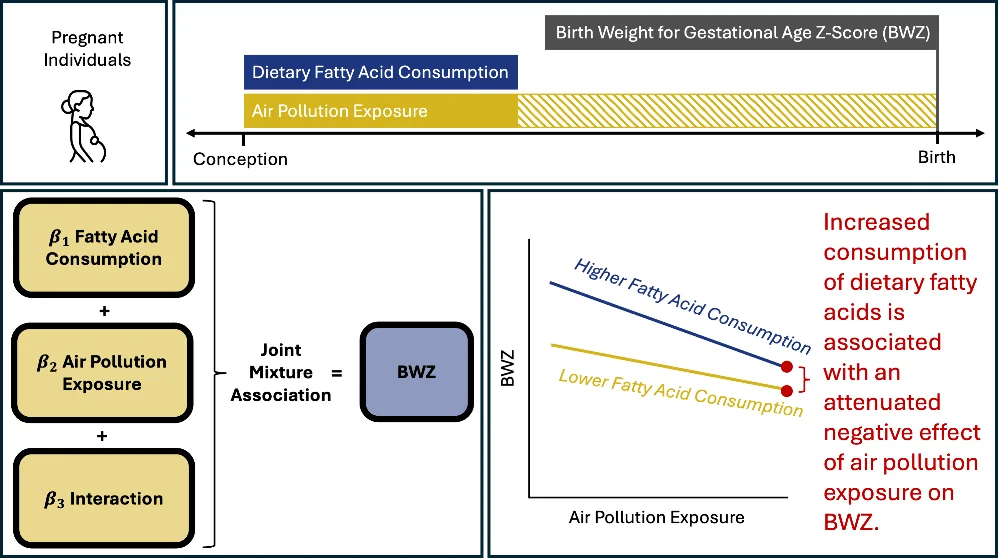

## Introduction

Low birth weight remains more prevalent among children born to Black and African American women in the United States (US) than among any other racial and ethnic group, and this disparity persists across education and socioeconomic stata.^1^ Adverse birth weight outcomes are associated with elevated risk of chronic disease later in life, supporting the notion that disparities originating in early life health may compound across the lifespan.^2^ Improving fetal growth among populations experiencing disproportionate risk, particularly racial and ethnic minorities in the US, represents an important opportunity to reduce longstanding health disparities.

Systematic reviews and meta-analyses link prenatal exposure to traffic-related air pollutants (TRAPs) such as carbon monoxide (CO), fine particulate matter (PM_2.5_), and nitrogen oxides (NO_*x*_), to adverse birth outcomes, including lower birth weight and increased risk of preterm birth with varying consistency.^3–5^ TRAP exposure may affect the placenta, leading to oxidative stress, inflammation, endocrine disruption, epigenetic changes, and vascular dysregulation, ultimately influencing birthweight.^6^

Dietary polyunsaturated fatty acids (PUFAs) are essential for healthy pregnancy and fetal development, and play key roles in mediating inflammation, fetal histone modification, DNA methylation, miRNA regulation, and placental angiogenesis.^7–9^ Certain PUFAs, particularly omega-3 fatty acids, may counteract oxidative stress and inflammatory pathways implicated in TRAP toxicity, suggesting that maternal PUFA intake during pregnancy could mitigate the adverse effects of TRAP exposure on fetal growth.

Maternal diet is a modifiable factor with intergenerational health implications. The Dietary Guideline Advisory Committee recommends tailored nutritional intake during pregnancy to support maternal and fetal health.^10,11^ Diets rich in fruits, vegetables, whole grains, nuts, legumes, seeds, and seafood, and low in red and processed meat, and fried foods, are associated with a lower risk of preterm birth.^12^ However, evidence linking prenatal diet and birth weight outcomes remains limited and insufficient.

Few studies have examined the joint associations of prenatal air pollution exposure and dietary factors on birth outcomes.^13–17^ Only two studies assessed dietary fats, and neither evaluated individual PUFA species.^13,17^ Moreover, limited research focuses on Black and African Americans, who face disproportionately higher TRAP exposure and the highest rates of preterm birth and low birth weight in the US.^18,19^ While assessing individual nutrients may appear reductionist, evaluating dietary components with well-characterized physiological effects may help elucidate biologically plausible, modifiable pathways underlying environmental effects on fetal growth. Such knowledge is critical for developing targeted public health and clinical interventions to mitigate the negative effects of TRAP exposure and reduce disparities in adverse birth outcomes. Therefore, we investigated the joint mixture associations of prenatal exposure to CO, PM_2.5_, and NO_*x*_ and maternal intake of total and PUFAs on birth weight z-scores (BWZ) in a cohort of pregnant African American individuals.

## Methods

### Study Participants

Participants were drawn from the Atlanta African American Maternal-Child Cohort (ATL AA), a socioeconomically diverse, prospective birth cohort that enrolls pregnant women seeking prenatal care at Emory Healthcare and Grady Health systems in Atlanta, GA, between 2014 and 2018. Eligibility criteria include self-identifying as non-Hispanic Black or African American, born in the US, aged between 18 and 40, singleton gestation, and absence of chronic medical conditions and chronic medication use.^20,21^ The analytic sample (*N* = 297) included participants with available prenatal dietary intake data and air pollution exposure assessment (Figure S1, Table S1). Written informed consent was obtained from all participants. The study was approved by the Institutional Review Board at Emory University (approval number 68441).

### Air Pollution Exposure Assessment

Residential ambient exposure to CO, PM_2.5_, and NO_*x*_ was estimated using a well-established TRAP pollutant model.^22^ Briefly, the model combined a calibrated RLINE-source dispersion model (250 × 250 m^2^ resolution) with downscaled bias-corrected outputs from the CMAQ model (12 km resolution), estimating daily ambient TRAP concentrations for metropolitan Atlanta (2002–2018, detailed description provided in the Supporting Information).

Individual-level exposure was determined using geocoded residential address at the first prenatal visit (8–14 weeks gestation). TRAP exposure was assessed for two gestational periods (the first trimester and the full pregnancy), as both may influence birth weight.^23–25^ Given inconsistent findings regarding a critical window of susceptibility, we considered both periods.^23,24^ The first trimester was of particular interest due to heighted vulnerability of the fetus to physiological and morphological abnormalities during this early period.^25^ This exposure period also coincided with the period that the dietary assessment data, explained later, aimed to estimate.

Conception and gestation timelines were calculated from the estimated date of confinement using standard clinical criteria.^26^ Exposure during each gestational period was defined as the mean daily exposure from the calculated conception date, which was determined by subtracting 266 days (38 weeks) from the estimated due date, to either the end of the twelfth week of gestation (first trimester) or date of birth (full pregnancy).

### Dietary Assessment

Participants completed a modified Block-Bodnar Food Frequency Questionnaire (FFQ) at two visits (8–14 and 24–30 weeks) to assess diet over the prior three months, with a focus on PUFA intake.^21^ FFQ data from the second visit were used to represent dietary intake during the gestational period when fetal growth is more variable.^27,28^ We did not observe a significant difference in PUFA intake values among those who had data available from visit 1 (*N* = 174) and visit 2 (*N* = 123) for the 11 PUFAs that consumption was estimated for (Table S2). Therefore, if FFQ data were available from the first visit but not the second, then these data were used in place (*N* = 262, 45.3%). Participants with implausible caloric intake (<500 or ≥ 5000 kcal/day, *N* = 40, 6.9%) were excluded.^29^ We estimated intake of linoleic acid (LA; 18:2n-6), α-linolenic acid (ALA; 18:3n-3), stearidonic acid (SA; 18:4n-3), arachidonic acid (AA; 20:4n-6), eicosapentaenoic acid (EPA; 20:5n-3), docosapentaenoic acid (DPA; 22:5n-3), docosahexaenoic acid (DHA; 22:6n-3), total n-6, total n-3, total monounsaturated fatty acid (MUFA), and total PUFA intake continuously as grams/day (see details in the Supporting Information). The MUFA:PUFA and n-6:n-3 ratios were calculated *post hoc*. All values except for ratios were log_2_-transformed to account for a potential log–linear relationship between fatty acid consumption and BWZ.^30^ Implausible values (±1.5 IQR) were removed (*N* = 4) to address information bias introduced from inaccurate estimates of nutrient intakes from meal portion sizes and components.^31^

### Outcome Assessment

The outcome of interest was sex-specific birth weight for gestational age z-score (BWZ). Birth weight in grams was abstracted from the medical record based on the first weight measured in the delivery room by the delivery room attendant. Gestational age at birth was determined from the delivery record using the best obstetrical estimate based upon the date of delivery in relation to the estimated date of confinement established by the 8–14 week prenatal visit.^26^ All participants received early pregnancy dating by last menstrual period (LMP) and/or early ultrasound, given enrollment criteria.^21^ BWZ were sex-specific and were estimated using a US population-based reference for singleton births.^32^

### Statistical Analysis

Linear regression was used to assess the joint mixture associations of single TRAPs and PUFAs on BWZ. The joint mixture association was considered the overall combined effect of both independent exposures and their interaction term for TRAPs and PUFAs on BWZ (see details in supplement). Because we were interested in how PUFA intake and TRAP exposure together shape birthweight outcomes, we treated both exposures as having equal causal standing. We therefore referred to this statistical interaction contributing to a joint mixture association rather than being an effect measure modification.^33^ These associations were interpreted as the effect of an interquartile range (IQR) increase of TRAP on BWZ when PUFA consumption doubled. Model fit was compared using likelihood ratio tests. We present both raw *p*-values and false discovery rate (FDR) corrected *q*-values estimated using the Benjamini-Hochberg method. TRAPs were scaled by IQR for comparability, and PUFAs were log_2_-transformed and centered at the mean. Models were adjusted for maternal age at enrollment, education (less than high school, high school, some college, college graduate or more), body mass index (BMI; kg/m^2^), caloric intake (kcal), and conception season (winter, spring, summer, fall). Seasons were defined using Atlanta’s weather patterns. Age, BMI, and caloric intake were truncated at the 2.5th and 97.5th percentiles and centered at the mean to minimize positivity assumption violations. Covariates were selected based on a directed acyclic graph (Figure S2).

The marginal joint associations of PUFA consumption and TRAP exposure on BWZ were visualized using splice plots. BWZ was predicted at each incremental value (10 parts per billion for CO and NO_*x*_, 0.1 μg/m^3^ for PM_2.5_) between the lowest and highest observed TRAP exposures when PUFA consumption was fixed at three different levels: the mean observed consumption, double the mean consumption, and half the mean consumption. Joint associations involving PUFA ratio values were not visualized since these were not log_2_-transformed. All other covariates, including dummy variables, were held constant at their mean values. The 25th, 50th, and 75th percentiles of TRAP exposure were labeled on the *x*-axes to highlight what effect an IQR increase in TRAP exposure has on BWZ at these three PUFA consumption levels.

Sensitivity analyses were conducted to evaluate the robustness of our analyses. Specifically, we examined low birth weight (≤37 weeks and <2500g) and small for gestational age (<10th percentile) to further evaluate relationships between TRAP exposures and fetal growth using modified Poisson regression with robust standard errors. All analyses used R (version 4.2.1).

## Results

A total of 297 participants were included in this analysis (Table 1). The mean age at enrollment was 25 years (standard deviation (SD)=5), with a mean early pregnancy BMI of 28 kg/m^2^ (SD = 7) and an average daily caloric intake of 1977 kcal (SD = 1005). Most conceptions occurred in summer (*N* = 94; 32%), with the fewest in winter (*N* = 51; 17%). Nearly half had some college education or more (*N* = 139; 47%).

**Table 1:** Characteristics of the 297 Study Participants^a^

Characteristic	*N* = 297
Daily Caloric Intake (kcal)	1,977 (1,005)
Body Mass Index (kg/m^2^)	28 (7)
Age (years)	25 (5)
Educational Attainment	
College Graduate or More	54 (18%)
Some College	85 (29%)
High School	111 (37%)
Less Than High School	47 (16%)
Conception Season	
Winter (Dec-Jan-Feb)	51 (17%)
Spring (Mar-Apr-May)	63 (21%)
Summer (Jun-Jul-Aug)	94 (32%)
Fall (Sep-Oct-Nov)	89 (30%)
BWZ^a^	–0.55 (1.01)
SGA^b^	76 (26%)
Term LBW^c^	23 (7.7%)
Supplemental n-3 PUFA intake	14 (4.7%)
Supplemental n-6 PUFA intake	14 (4.7%)

a Continuous variables are reported as mean (standard deviation), and categorical variables are reported as *N* (%).

b Sex-specific birth weight for gestational age z-score.

c Small for gestational age.

d Term low birth weight

TRAP exposure distributions were similar across the first trimester and full pregnancy (Table 2). Median first-trimester CO exposure was 683.18 ppb (IQR = 269.64) and PM_2.5_ was 9.02 mg/m^3^ (IQR = 1.84). Median NO_*x*_ exposure was 62.01 ppb (IQR = 34.42) for the first trimester. Correlations between CO and NO_*x*_ were high (r = 0.90–0.98), while PM_2.5_ showed moderate correlation with CO and NO_*x*_ (r = 0.26–0.58) (Table S3).

**Table 2:** Traffic-Related Air Pollution (TRAP) Exposure and Dietary Polyunsaturated Fatty Acid (PUFA) Consumption Summaries^a^

		Min	Q1	Median	Q3	Max
Air Pollutants						
CO	T1	278.83	579.14	683.18	848.78	2643.13
	Full	315.80	586.49	689.56	857.18	2721.12
NO_*x*_	T1	12.51	47.58	62.01	82.00	240.43
	Full	18.18	49.55	62.36	79.04	266.74
PM_2.5_	T1	6.54	8.22	9.02	10.06	13.19
	Full	7.16	8.36	8.83	9.39	13.09
Fatty Acids						
LA		2.77	8.12	12.28	17.80	39.80
ALA		0.27	0.85	1.30	1.90	5.26
SA		0.0	0.001	0.002	0.0049	0.04
AA		0.01	0.06	0.11	0.16	0.53
EPA		0.0007	0.006	0.01	0.03	0.23
DPA		0.0	0.003	0.006	0.01	0.06
DHA		0.001	0.02	0.03	0.07	0.34
Total n-6		2.79	8.14	12.43	17.95	39.98
Total n-3		0.27	0.93	1.39	2.02	5.31
MUFA		6.19	18.67	27.78	42.07	88.74
PUFA		3.08	9.74	14.72	21.43	46.02
MUFA:PUFA		0.96	1.66	1.87	2.10	3.31
n-6:n-3		5.29	8.10	9.06	9.97	16.84

a TRAP exposure was estimated using data from an advanced machine learning model with 250 × 250 m^2^ spatial resolution that was geocoded to the participants’ residential addresses. Carbon monoxide (CO) and nitrogen oxides (NO_*x*_) exposure are reported in parts per billion (ppb). Fine particulate matter (PM_2.5_) exposure is reported in μg/m^3^. A food frequency questionnaire was used to estimate linoleic acid (LA; 18:2n-6), α-linolenic acid (ALA; 18:3n-3), stearidonic acid (SA; 18:4n-3), arachidonic acid (AA; 20:4n-6), eicosapentaenoic acid (EPA; 20:5n-3), docosapentaenoic acid (DPA; 22:5n-3), docosahexaenoic acid (DHA; 22:6n-3), total n-6, total n-3, total monounsaturated fatty acid (MUFA), and total PUFA intake continuously as g/day. The MUFA:PUFA and n-6:n-3 ratios were calculated *post hoc*. TRAP exposure was similar during the first trimester of pregnancy (T1) and the full gestational period (Full). n-6 PUFAs were consumed more than n-3, and LA was the most consumed single PUFA.

Participants’ PUFA consumption varied widely across participants (Table 2). Median daily consumption was greatest for LA at 12.3 g (IQR = 9.73) and lowest for SA at 0.002 g (IQR = 0.004). Few participants used dietary n-3 supplements (4.7%) or n-6 supplements (4.7%) (Table 1). Therefore, the variation in estimated PUFA consumption seems primarily due to differences in dietary intake. Total n-6 consumption (12.43 g/day, IQR = 9.82) was higher than n-3 intake (1.39 g/day, IQR = 1.08). Consumption of LA was highly correlated with ALA (r = 0.95) (Table S4). Given that LA and ALA were the highest consumed n-6 and n-3 PUFAs, respectively, total n-6 and total n-3 PUFA consumption were highly correlated (r = 0.94) (Tables 2, S4). Total MUFA consumption was also highly correlated with total PUFA consumption (r = 0.95) (Table S4).

Significant interaction and joint mixture associations were observed between PM_2.5_ and both LA and ALA during the first trimester (Table 3). When LA was held at its mean value, an IQR increase in PM_2.5_ was associated with a 0.08 (95% CI: −0.17, 0.02) unit decrease in BWZ. In contrast, when LA intake was doubled, the same PM_2.5_ increase was associated with a 1.09 (95% CI: 0.17, 2.02, *p*-int = 0.09) unit increase in BWZ. Splice plots showed that greater LA intake was associated with greater BWZ across levels of PM_2.5_ exposure, and increased PM_2.5_ exposure was associated with a decrease in BWZ across levels of LA consumption (Figure 1c). While the association of an IQR increase in PM_2.5_ exposure on BWZ was more dramatic among those who consumed more LA, BWZ remained higher among those who consumed double the mean LA value compared to the mean value.

**Figure 1:**
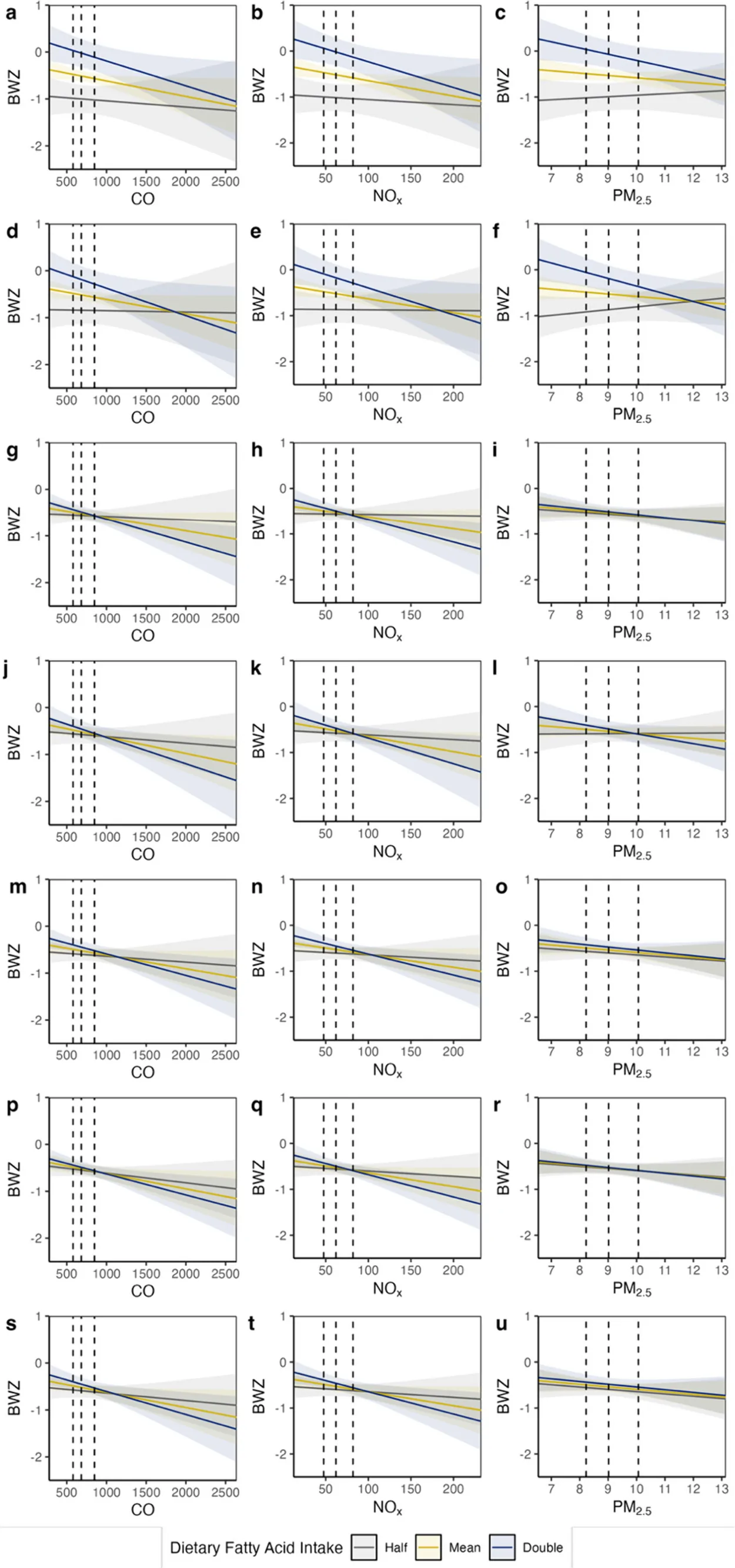
Splice plots showing the marginal dose–response relationship between average daily traffic-related air pollution (TRAP) exposure during the first trimester and birth weight z-score (BWZ) at varying levels of categorized dietary fatty acid intake during early pregnancy. Shaded regions represent 95% confidence intervals. BWZ values were predicted across observed TRAP exposure values, and the vertical dashed lines represent the 25th, 50th, and 75th percentile of observed TRAP exposure. PUFAs: linoleic acid (LA; 18:2n-6), α-linolenic acid (ALA; 18:3n-3), stearidonic acid (SA; 18:4n-3), arachidonic acid (AA; 20:4n-6), eicosapentaenoic acid (EPA; 20:5n-3), docosapentaenoic acid (DPA; 22:5n-3), docosahexaenoic acid (DHA; 22:6n-3). TRAPs: carbon monoxide (CO), nitrogen oxides (NO_*x*_), fine particulate matter (PM_2.5_). a) CO*LA, b) NO_x_*LA, c) PM_2.5_*LA, d) CO*ALA, e) NO_x_*ALA, f) PM_2.5_*ALA, g) CO*SA, h) NO_x_*SA, (i) PM_2.5_*SA, j) CO*AA, k) NO_x_*AA, l) PM_2.5_*AA, m) CO*EPA, n) NO_x_*EPA, o) PM_2.5_*EPA, p) CO*DPA, q) NO_x_*DPA, r) PM_2.5_*DPA, s) CO*DHA, t) NO_x_*DHA and u) PM_2.5_*DHA.

**Table 3:** Joint Mixture Associations of a Doubling of Individual Dietary Polyunsaturated Fatty Acid (PUFA) Intake during Early Pregnancy and Interquartile Range (IQR) Change of the Average Daily Traffic-Related Air Pollution (TRAP) during the First Trimester on Birth Weight z-Score (BWZ) Adjusted for Average Daily Caloric Intake, Body Mass Index at Recruitment, Age at Recruitment, Educational Attainment, and Conception Season^a^,^b^

		PUFA	TRAP	PUFA*TRAP	Joint Mixture	*p-*int	*q*-int
LA	CO	0.61 (0.19, 1.04)*	–0.05 (−0.11, 0)*	–0.03 (−0.1, 0.04)	0.53 (0.14, 0.92)*	0.35	0.46
	NO_*x*_	0.62 (0.22, 1.02)*	–0.07 (−0.13, 0)*	–0.04 (−0.12, 0.04)	0.51 (0.16, 0.86)*	0.32	0.45
	PM_2.5_	1.26 (0.26, 2.26)*	–0.08 (−0.17, 0.02)	–0.09 (−0.2, 0.02)	1.09 (0.17, 2.02)*	**0.09**^	0.27
ALA	CO	0.51 (0.07, 0.94)*	–0.05 (−0.1, 0)	–0.04 (−0.12, 0.03)	0.41 (0, 0.82)	0.24	0.39
	NO_*x*_	0.5 (0.1, 0.91)*	–0.06 (−0.12, 0)	–0.05 (−0.14, 0.04)	0.39 (0.02, 0.76)*	0.25	0.39
	PM_2.5_	1.4 (0.41, 2.39)*	–0.08 (−0.18, 0.02)	–0.12 (−0.23, −0.02)*	1.2 (0.27, 2.13)*	**0.02**^	0.26
SA	CO	0.17 (0.03, 0.32)*	–0.05 (−0.1, 0.01)	–0.03 (−0.06, 0)*	0.09 (−0.05, 0.23)	**0.03**^	0.26
	NO_*x*_	0.17 (0.04, 0.31)*	–0.05 (−0.12, 0.01)	–0.05 (−0.08, −0.01)*	0.08 (−0.04, 0.19)	**0.01**^	**0.20** ^**#**^
	PM_2.5_	0.13 (−0.26, 0.51)	–0.07 (−0.17, 0.03)	–0.01 (−0.06, 0.03)	0.04 (−0.28, 0.36)	0.6	0.69
AA	CO	0.2 (−0.08, 0.47)	–0.06 (−0.11, −0.01)*	–0.03 (−0.08, 0.02)	0.1 (−0.13, 0.34)	0.19	0.37
	NO_*x*_	0.18 (−0.06, 0.43)	–0.07 (−0.13, 0)*	–0.04 (−0.1, 0.02)	0.08 (−0.12, 0.27)	0.16	0.37
	PM_2.5_	0.57 (−0.19, 1.33)	–0.07 (−0.17, 0.03)	–0.06 (−0.15, 0.02)	0.44 (−0.2, 1.08)	0.16	0.37
EPA	CO	0.19 (0.03, 0.34)*	–0.05 (−0.1, 0)	–0.03 (−0.06, 0)	0.11 (−0.03, 0.25)	**0.07**^	0.27
	NO_*x*_	0.18 (0.05, 0.32)*	–0.06 (−0.12, 0.01)	–0.03 (−0.07, 0)*	0.09 (−0.03, 0.21)	**0.04**^	0.26
	PM_2.5_	0.16 (−0.28, 0.59)	–0.07 (−0.17, 0.03)	–0.01 (−0.06, 0.04)	0.08 (−0.29, 0.44)	0.65	0.72
DPA	CO	0.11 (−0.02, 0.23)	–0.05 (−0.11, 0)*	–0.02 (−0.04, 0)	0.03 (−0.12, 0.19)	**0.07**^	0.27
	NO_*x*_	0.14 (0.02, 0.26)*	–0.06 (−0.12, 0)	–0.04 (−0.06, −0.01)*	0.04 (−0.08, 0.17)	**0.01**^	**0.20** ^**#**^
	PM_2.5_	0.09 (−0.36, 0.54)	–0.07 (−0.17, 0.03)	–0.01 (−0.06, 0.04)	0.01 (−0.51, 0.53)	0.7	0.76
DHA	CO	0.18 (0, 0.35)	–0.05 (−0.11, 0)*	–0.03 (−0.06, 0.01)	0.1 (−0.06, 0.25)	0.11	0.31
	NO_*x*_	0.17 (0.01, 0.33)*	–0.06 (−0.12, 0)	–0.03 (−0.07, 0)	0.08 (−0.06, 0.21)	**0.08**^	0.27
	PM_2.5_	0.11 (−0.42, 0.63)	–0.07 (−0.17, 0.03)	–0.01 (−0.07, 0.05)	0.03 (−0.42, 0.48)	0.83	0.87

a *p < 0.05, ^*p*-int < 0.1, ^#^ *q*-int < 0.2.

b PUFAs: linoleic acid (LA; 18:2n-6), a-linolenic acid (ALA; 18:3n-3), stearidonic acid (SA; 18:4n-3), arachidonic acid (AA; 20:4n-6), eicosapentaenoic acid (EPA; 20:5n-3), docosapentaenoic acid (DPA; 22:5n-3), docosahexaenoic acid (DHA; 22:6n-3). TRAPs: carbon monoxide (CO), nitrogen oxides (NO_*x*_), fine particulate matter (PM_2.5_)

Similarly, when ALA was held at its mean value, an IQR increase in PM_2.5_ was associated with a 0.08 (95% CI: −0.18, 0.02) unit decrease in BWZ. When ALA intake was doubled, an IQR increase in PM_2.5_ was associated with a 1.20 (95% CI: 0.27, 2.13, *p*-int = 0.02) unit increase in BWZ. Splice plots similarly showed that greater ALA intake was associated with greater BWZ across levels of PM_2.5_ exposure, and increased PM_2.5_ exposure was associated with a decrease in BWZ across levels of ALA consumption (Figure 1f). BWZ remained higher among those who consumed double the mean ALA value compared to the mean value, even after the effect of an IQR increase in PM_2.5_ exposure. A similar joint mixture association was noted between ALA and PM_2.5_ on BWZ (β_TRAP_=-0.09, β_joint_ = 1.98, *p*-int = 0.02) during the full pregnancy period (Table S5).

Among the categorized fatty acids, significant interaction and joint mixture associations were observed for total n-6, n-3, MUFA, and PUFA with PM_2.5_ during the first trimester (Table 4). When n-6 PUFA was held at its mean value, an IQR increase in PM_2.5_ was associated with a 0.08 (95% CI: −0.17, 0.02) unit decrease in BWZ. When n-6 PUFA intake was doubled, the same increase in PM_2.5_ was associated with a 1.09 (95% CI: 0.16, 2.02, *p*-int = 0.09) unit increase in BWZ. When n-3 PUFA was held at its mean value, an IQR increase in PM_2.5_ was associated with a 0.08 (95% CI: −0.18, 0.02) unit decrease in BWZ. When the intake of n-6 PUFA was doubled, the same increase in PM_2.5_ was associated with a 1.12 (95% CI: 0.19, 2.05, *p*-int = 0.04) unit increase. When MUFA was held at its mean value, an IQR increase in PM_2.5_ was associated with a 0.07 (95% CI: −0.17, 0.03) unit decrease in BWZ. When the intake of MUFA was doubled, the same increase in PM_2.5_ was associated with a 0.99 (95% CI: 0.03, 1.96, *p*-int = 0.09) unit increase. Finally, when PUFA was held at its mean value, an IQR increase in PM_2.5_ was associated with a 0.07 (95% CI: −0.17, 0.02) unit decrease in BWZ. When the amount of PUFA was doubled, the same increase in PM_2.5_ was associated with a 1.11 (95% CI: 0.17, 2.05, *p*-int = 0.09) unit increase.

**Table 4:** Joint Mixture Associations of a Doubling of Categorized Dietary Polyunsaturated Fatty Acid (PUFA) Intake during Early Pregnancy and Interquartile Range (IQR) Change of the Average Daily Traffic-Related Air Pollution (TRAP) during the First Trimester on Birth Weight z-Score (BWZ) Adjusted for Average Daily Caloric Intake, Body Mass Index at Recruitment, Age at Recruitment, Educational Attainment, and Conception Season^a^

		PUFA	TRAP	PUFA*TRAP	Joint Mixture	*p*-int	*q*-int
Total n-6	CO	0.61 (0.18, 1.04)*	–0.05 (−0.11, 0)*	–0.03 (−0.1, 0.04)	0.53 (0.13, 0.92)*	0.35	0.46
	NO_*x*_	0.62 (0.22, 1.02)*	–0.07 (−0.13, 0)*	–0.04 (−0.12, 0.04)	0.51 (0.16, 0.86)*	0.32	0.45
	PM_2.5_	1.26 (0.26, 2.26)*	–0.08 (−0.17, 0.02)	–0.09 (−0.2, 0.02)	1.09 (0.16, 2.02)*	**0.09**^	0.27
Total n-3	CO	0.53 (0.1, 0.96)*	–0.05 (−0.1, 0)	–0.05 (−0.12, 0.03)	0.43 (0.03, 0.84)*	0.2	0.37
	NO_*x*_	0.54 (0.14, 0.95)*	–0.06 (−0.12, 0)	–0.06 (−0.15, 0.03)	0.42 (0.06, 0.78)*	0.17	0.37
	PM_2.5_	1.31 (0.3, 2.31)*	–0.08 (−0.18, 0.02)	–0.11 (−0.22, 0)*	1.12 (0.19, 2.05)*	**0.04**^	0.26
MUFA	CO	0.5 (0.03, 0.96)*	–0.05 (−0.1, 0)	–0.04 (−0.11, 0.03)	0.41 (−0.04, 0.85)	0.27	0.41
	NO_*x*_	0.49 (0.06, 0.92)*	–0.06 (−0.12, 0)	–0.05 (−0.14, 0.03)	0.38 (−0.03, 0.78)	0.24	0.39
	PM_2.5_	1.16 (0.09, 2.23)*	–0.07 (−0.17, 0.03)	–0.1 (−0.21, 0.02)	0.99 (0.03, 1.96)*	**0.09**^	0.39
PUFA	CO	0.66 (0.22, 1.1)*	–0.05 (−0.1, 0)	–0.04 (−0.11, 0.03)	0.57 (0.17, 0.96)*	0.25	0.37
	NO_*x*_	0.67 (0.25, 1.08)*	–0.06 (−0.13, 0)*	–0.05 (−0.14, 0.03)	0.55 (0.2, 0.9)*	0.2	0.27
	PM_2.5_	1.28 (0.26, 2.29)*	–0.07 (−0.17, 0.02)	–0.09 (−0.2, 0.02)	1.11 (0.17, 2.05)*	**0.09**^	0.27
MUFA:PUFA	CO	–0.3 (−1.2, 0.59)	–0.06 (−0.38, 0.26)	0 (−0.17, 0.17)	–0.36 (−1.33, 0.61)	1	1
	NO_*x*_	–0.34 (−1.08, 0.4)	–0.08 (−0.43, 0.27)	0.01 (−0.18, 0.2)	–0.41 (−1.27, 0.44)	0.93	0.95
	PM_2.5_	–0.99 (−3.3, 1.33)	–0.23 (−0.72, 0.27)	0.08 (−0.18, 0.34)	–1.14 (−3.53, 1.26)	0.57	0.67
n-6:n-3	CO	–0.01 (−0.14, 0.12)	–0.15 (−0.38, 0.07)	0.01 (−0.01, 0.03)	–0.15 (−0.4, 0.09)	0.39	0.49
	NO_*x*_	–0.01 (−0.14, 0.13)	–0.17 (−0.48, 0.13)	0.01 (−0.02, 0.04)	–0.17 (−0.48, 0.14)	0.46	0.56
	PM_2.5_	–0.31 (−0.77, 0.14)	–0.44 (−0.92, 0.05)	0.04 (−0.01, 0.09)	–0.71 (−1.54, 0.11)	0.13	0.34

a PUFAs were log_2_-transformed except for ratio values. *p < 0.05, ^*p*-int <0.1, #*q*-int <0.2. PUFAs: total n-6 polyunsaturated fatty acid (Total n-6), total n-3 polyunsaturated fatty acid (Total n-3), total monounsaturated fatty acid (MUFA) to total polyunsaturated fatty acid (PUFA) ratio, total n-6 polyunsaturated fatty acid to total n-3 polyunsaturated fatty acid ratio (n-6:n-3). TRAPs: carbon monoxide (CO), nitrogen oxides (NO_*x*_), fine particulate matter (PM_2.5_).

There was similarly a positive joint mixture association between total n-3 and PM_2.5_ (β_TRAP_=-0.09, β_joint_ = 2.01, *p*-int = 0.02) as well as total PUFA and PM_2.5_ (β_TRAP_=-0.09, β_joint_ = 1.62, *p*-int = 0.08) on BWZ during the full pregnancy period (Table S6). There was also a negative joint effect between increased MUFA:PUFA ratio and PM_2.5_ (β_TRAP_=-0.53, β_joint_=-4.42, *p*-int = 0.07) as well as increased n-6:n-3 ratio and PM_2.5_ (β_TRAP_=-0.53, β_joint_=-1.12, *p*-int = 0.02) on BWZ during the same exposure period (Table S6). Similar to what we observed with LA and ALA, we observed that PM_2.5_ was negatively associated with BWZ across consumption levels of either n-6 (Figure 2c) or n-3 (Figure 2f) PUFA, though BWZ was higher among those with greater consumption of PUFA.

**Figure 2:**
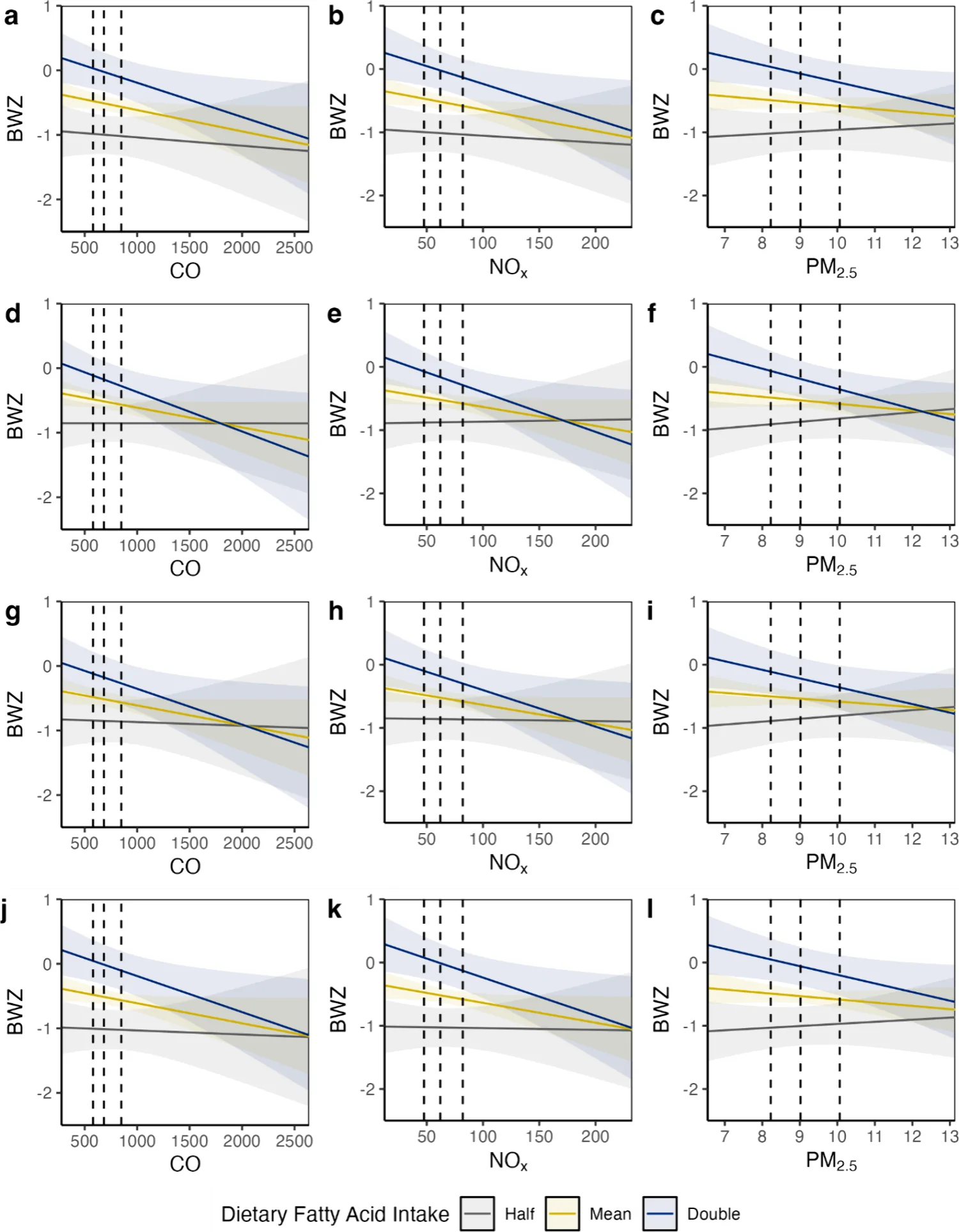
Splice plots showing the marginal dose–response relationship between average daily traffic-related air pollution (TRAP) exposure during the first trimester and birth weight z-score (BWZ) at varying levels of categorized dietary fatty acid intake during early pregnancy. Shaded regions represent 95% confidence intervals. BWZ values were predicted across observed TRAP exposure values, and the vertical dashed lines represent the 25th, 50th, and 75th percentile of observed TRAP exposure. PUFAs: total n-6 polyunsaturated fatty acid (Total n-6), total n-3 polyunsaturated fatty acid (Total n-3), total monounsaturated fatty acid (MUFA), total polyunsaturated fatty acid (PUFA). TRAPs: carbon monoxide (CO), nitrogen oxides (NO_*x*_), fine particulate matter (PM_2.5_). a) CO*n-6, b) NO_x_*n-6, c) PM_2.5_*n-6, d) CO*n-3, e) NO_x_*n-3, f) PM_2.5_*n-3, g) CO*MUFA, h) NO_x_*MUFA, (i) PM_2.5_*MUFA, j) CO*PUFA, k) NO_x_*PUFA and l) PM_2.5_*PUFA

Sensitivity analyses using small for gestational age and term low birth weight outcomes yielded inconsistent results, likely due to the small analytical sample size and low prevalence of these outcomes. However, negative marginal joint associations were noted for stearidonic acid (SA) and docosapentaenoic acid (DPA) in combination with NO_*x*_ exposure on these measures of fetal growth (Table S7–S9).

## Discussion

In this prospective birth cohort of socioeconomically diverse African American pregnant individuals, we identified positive joint mixture effects of TRAP exposure and dietary intake of LA, ALA, total n-6 PUFA, total n-3 PUFA, total MUFA, and total PUFA on BWZ. These joint associations were most pronounced for PM_2.5_ compared to CO and NO_*x*_, and they were observed consistently across the first trimester and full pregnancy periods. Because LA and ALA were the n-6 and n-3 PUFAs with the greatest consumption, respectively, the associations of total n-6 and total n-3 PUFA are likely driven by these individual components. Together, these findings suggest that dietary PUFAs and TRAP exposure may share physiological pathways that influence fetal growth.

Previous work in the ATL AA cohort has similarly demonstrated that elevated TRAP exposure is associated with adverse fetal growth outcomes. Increased PM_2.5_ exposure was positively associated with the odds of preterm birth, and the effect of an IQR increase during the first trimester exposure period was similar in magnitude to that of an IQR increase during the full pregnancy period.^34^ A metabolomics study found that perturbations in protein digestion and absorption and aromatic amino acid metabolism were associated with both PM_2.5_ exposure and preterm and early term birth.^35^

To the best of our knowledge, no other studies have examined the interactive effect of PUFAs and TRAP on BWZ. Only one cross-sectional study of 684 participants assessed dietary fat (e.g., dichotomized caloric intake, percent of dietary calories contributed to total fat, and percent of dietary calories contributed to saturated fat) as a modifier of ambient air pollution exposure and preterm birth, but it showed weak evidence of interaction.^13^ Another cross-sectional study of 481 participants explored fish consumption and PM_2.5_ in relation to birth weight, finding no statistical interaction despite a potential protective trend with higher fish intake.^17^

Studies of other child health outcomes have shown that there may be meaningful joint associations of TRAP and PUFA or effect measure modifications of TRAP by PUFA. A prospective birth cohort of 657 pregnant women and their children found that the association between prenatal exposure to PM_2.5_ and incident childhood allergic rhinitis was modified by maternal erythrocyte PUFA levels where those who experienced greater PUFA exposure were at lower risk of developing respiratory allergy outcomes.^36^ Similarly, a birth cohort with 20,730 participants found that the joint effect of beneficial nutritional additives, including maternal intake of the PUFA DHA, and the outdoor air pollutants NO_2_, O_3_, and PM_2.5_ on the prevalence of childhood food allergies was attenuated compared to the effect of these pollutants alone.^37^ A birth cohort of 126 pregnant women found that, using Bayesian kernel machine regression, there was little evidence of a joint association between outdoor air pollutants and prenatal nutrients, including n-3 and n-6 PUFAs.^38^

Mechanistically, TRAP exposure is known to induce oxidative stress and systemic inflammation, disrupting maternal and fetal health.^6^ Air pollutants may contain free radicals themselves or may generate secondary oxidants by interacting with lipids and proteins in the lung lining fluid compartment. This oxidative challenge brings the redox state of the pulmonary environment out of homeostasis, and endogenous antioxidant systems cannot neutralize the threat. The resulting reactive oxygen and nitrogen species may alter or damage DNA, proteins, lipids, and carbohydrate structures. The later trimesters of pregnancy are already associated with an increase in oxidative stress, so an additional oxidative burden caused by TRAP exposure could overwhelm the physiological mechanisms that seek to neutralize the oxidative species.^39^ Additionally, these exposures could lead to an increase in global maternal inflammation, epigenetic alterations, and endocrine disruption.

PUFAs, particularly LA and ALA and their downstream metabolites, play central roles in mediating inflammatory processes and may therefore attenuate the TRAP-related oxidative and inflammatory stress. Supporting this hypothesis, a randomized controlled trial of fish oil supplementation in healthy college students found interactions between the percent change in the relative concentration of 11 annotated fatty acid metabolites (including n-3 PUFAs, n-6 PUFAs, n-9 PUFAs, and AA derivatives) and a 10 mg/m^3^ increase in PM_2.5_ exposure, with decreases in pro-inflammatory metabolites observed at higher PUFA intake levels.^40^ We similarly identified a consistent pattern where increased consumption of LA and ALA, precursors to long-chain polyunsaturated fatty acids involved in anti-inflammatory pathways, was positively associated with fetal growth within a given range of TRAP exposure.^41^ This interpretation is further supported by a cross-sectional untargeted metabolomics analysis of maternal serum finding that high exposure to CO, NO_*x*_, and PM_2.5_ was associated with a significant decrease in the relative abundance of LA and significant modulation of the LA metabolism pathway.^42^ Future mechanistic studies are needed to explore how lipid metabolism alterations may mediate the observed effects.

This study has multiple strengths. First, it addresses a key gap in the literature using a birth cohort with well-characterized TRAP exposure, dietary intake, and birth outcome data. Second, our outcome ascertainment methods minimize the possibility of inaccurate measurement of birth weight and gestational age at birth since measures were abstracted from medical records by trained medical personnel, minimizing misclassification of birth outcomes. The outcome of interest in this study, BWZ, is an established marker of fetal growth of clinical and public health significant that is linked with an increased risk of cardiometabolic disease later in life.^43,44^ Specifically, a low birth weight is associated with altered fatty acid metabolism, impaired glucose regulation, type 2 diabetes mellitus, metabolic syndrome, and arterial hypertension in adulthood, suggesting that the impact of prenatal air pollution exposure on pregnancy can have long lasting effects on cardiometabolic health across a child’s lifespan.^6,7^ Our study participants were also exclusively African American, a population significantly underrepresented in environmental epidemiologic studies despite facing the ’double jeopardy’ of multiple environmental stressors that exacerbate existing health disparities and environmental injustice. These findings may provide insights into developing precision nutrition interventions as an effective way to attenuate the persistent health disparities in the elevated TRAP exposure and the corresponding risk of adverse birth outcomes within the African American population.

There are limitations to discuss as well. First, many statistical tests were conducted which inflates the likelihood of false positive results. Because the primary objective of the ATL AA cohort is to identify contributing factors to adverse birth outcomes in pregnant Black and African American people, participants were excluded if they had a current diagnosis of any chronic medical condition or had any use of chronic prescription medications. This may result in a loss of generalizability. Because the cohort was not powered to investigate interaction, the resulting sample size may be too small to fully capture these effects. Due to the study design, our results do not necessarily imply a causal relationship. These observed alterations could potentially result from coexposure confounding, which should be investigated in future mixture analyses. TRAP exposure was estimated solely based on the residential addresses of the cohort participants. Ignoring occupational mobility and commuting modes of pregnant women could lead to mismeasurement of air pollution exposure. Critical TRAP exposure periods during pregnancy are also not fully understood, so there is a need to evaluate these associations over the course of additional intervals. TRAP exposure levels in this study were broadly representative of urban US air pollution, though our NO_*x*_ estimates were higher and CO levels lower than national averages.^45^ Similarly, PUFA intake was higher than in general populations (i.e., NHANES 2015–2016),^46^ possibly reflecting greater dietary awareness during pregnancy. Finally, there may be unmeasured confounding by overall maternal dietary pattern which could be addressed in future studies by calculating a dietary index score for study participants. This study used FFQ data as an estimate of PUFA exposure. While these instruments have been validated in pregnant study populations, they characterize PUFAs to varying degrees.^47,48^ Yet, because this was a prospective cohort, we do not anticipate that measurement error of air pollution or dietary intake would be differential according to birthweight, and therefore, any bias would be expected to be toward the null. This source of exposure measurement error, however, could have potentially diminished the power of the analysis.

While using a FFQ measure for fatty acid exposure is a limitation, multiple studies have demonstrated associations between internal measures of fatty acids and birth outcomes. A large prospective cohort study observed that higher maternal plasma concentration of omega-3 PUFA relative to omega-6 PUFA was associated with favorable fetal growth velocity, birth weight, and gestational length.^49^ Another longitudinal study investigating the effect of maternal erythrocyte concentration of PUFAs on birth weight found that greater amounts of AA were associated with lower weight-for-age z-score at 1 month, and there was no association with the omega-3 to omega-6 ratio.^50^ A metabolomics study of cord serum found that lower DHA and omega-3 were associated with increased BWZ, though the opposite relationship was observed for gestational age.^51^ Together, these findings suggest that there are statistical associations between internal measures of fatty acids with birth weight outcomes, though the observed direction of these associations may be unclear.

## Conclusions

Among pregnant African American individuals in an urban US setting, higher maternal intake of polyunsaturated fatty acids was associated with attenuation of the adverse association between traffic-related air pollution and fetal growth (BWZ). Since dietary intake is modifiable through public health and clinical practice interventions, these findings highlight the potential role of precision nutrition strategies in mitigating environmentally mediated disparities in adverse birth outcomes. Future research into the molecular mechanisms underlying TRAP and dietary macronutrient interactions are warranted to inform public health strategies and clinical practice.

## Supplementary Material



## Notes

Supplemental methods, flowchart for study participant inclusion, comparison of study participants between those included in the present analysis and those with complete birth outcome data, comparison of PUFA consumption estimates from two different study visits, directed acyclic graph, correlation matrices of TRAP and PUFA, tables and figures from sensitivity analyses using full pregnancy TRAP exposure, tables from sensitivity analyses that used small for gestational age and term low birth weight as outcomes (PDF)
