# Supplementary material for: Joint Mixture Associations of Prenatal Air Pollution and Polyunsaturated Fatty Acid Intake on Birth Weight Outcomes in an African American Cohort
Source: Environ Health (Wash). 2026 Apr 22;4(8):1637–47. doi: 10.1021/envhealth.5c00490 (PMC13504550; doi:10.1021/envhealth.5c00490)
Supplement: Supplementary file 1 [file eh5c00490_si_001.pdf]

Supporting Information

Joint Mixture Associations of Prenatal Air Pollution and Polyunsaturated Fatty Acid Intake on Birth Weight Outcomes in an African American Cohort

Benjamin R. Kessler,[1] Anne L. Dunlop,[1,2] Robert B. Hood,[3] Stephanie M. Eick,[1] Jiada James Zhan,[4] Susan S. Hoffman,[5], Zhihao Jin,[1] Youran Tan,[1] Erin Ferranti,[6] Jeremy Sarnat,[1] Audrey J. Gaskins,[5] Howard H. Chang,[7] Christine Ekenga,[1] Donghai Liang*[1]

1. Emory University Gangarosa Department of Environmental Health, Atlanta, GA, USA
2. Emory University School of Medicine Department of Gynecology & Obstetrics, Atlanta, GA, USA
3. Ohio State University, College of Public Health, Columbus, OH, USA
4. Emory University Nutrition & Health Sciences Doctoral Program, Atlanta, GA, USA
5. Emory University Department of Epidemiology, Atlanta, GA, USA
6. Emory University Nell Hodgson Woodruff School of Nursing, Department of Gynecology & Obstetrics, Atlanta, GA, USA
7. Emory University Department of Biostatistics & Bioinformatics, Atlanta, GA, USA

Corresponding Authors:

Donghai Liang, PhD
Associate Professor, Gangarosa Department of Environmental Health
Emory University Rollins School of Public Health
1518 Clifton Rd, Rm 7021
Atlanta, GA, USA
30322
donghai.liang@emory.edu

Anne L. Dunlop, MD, MPH
Professor, Department of Gynecology and Obstetrics
Emory University School of Medicine
101 Woodruff Circle, Rm 4333
Atlanta, GA, USA
30322
amlang@emory.edu

Supplemental Methods – Exposure Assessment

We estimated residential ambient exposure to CO, $PM_{2.5}$, and NO_x for each of the ATL AA cohort study participants using a well-established TRAP pollutant model.[20] Briefly, this modeling procedure involved two major steps. First, a calibrated Research LINE (RLINE)-source dispersion model was used to estimate annual-averaged traffic-related CO, $PM_{2.5}$, and NO_x at 250x250 m^2 spatial resolution. RLINE data were then used to downscale bias-corrected CO, $PM_{2.5}$, and NO_x outputs from the Community Multi-Scale Air Quality (CMAQ) model. CMAQ is a publicly available chemical transport model that simulates daily air pollution concentrations at a lower spatial resolution of 12 km.[20] The final air quality model, which incorporated comprehensive chemistry and emission sources, estimated daily ambient TRAP covering metropolitan Atlanta from 2002-2018 with a spatial resolution of approximately 250x250 m^2.

Individual exposures were estimated by geocoding participants' residential addresses at the time of the first prenatal visit (8-14 weeks of gestation) and linking this to daily ambient TRAP concentrations. There are conflicting findings as to whether there is a critical window of exposure during pregnancy that increases the risk for lower birth weight or BWZ.[21,22] Therefore, we considered TRAP exposure during the first trimester and the full pregnancy duration. The first trimester was investigated because of the susceptibility of the fetus to physiological and morphological abnormalities during this period.[23] This exposure period also coincided with the period that the dietary assessment data explained later aimed to estimate. First trimester TRAP exposure was defined as the average daily exposure from the presumed first day of last menstrual period through the end of the thirteenth week of gestation; the presumed first day of

menstrual period and date representing the end of the thirteenth week of gestation were calculated by subtracting 40 weeks (280 days) and 26 weeks and 1 day (183 days), respectively, from the estimated date of confinement, which was estimated from a combination of last menstrual period and 8-14 week ultrasound in accordance with standard clinical criteria.[24] Exposure during the duration of the full pregnancy was similarly calculated from the presumed first day of last menstrual period through the date of birth.

Supplemental Methods – Dietary Assessment

A modified Block-Bodnar semi-quantitative Food Frequency Questionnaire (FFQ) was administered to participants at two prenatal care visits between 8-14 and 24-30 weeks of gestation to assess dietary intake and supplementation over the previous three months, with a focus on ascertainment of vitamin D, folate, essential trace element, and PUFA intake.[19] These data were collected with NutritionQuestion's online FFQ and analyzed with the service's Data-on-Demand (https://www.nutritionquest.com/assessment/data-on-demand/). Data were available for 578 study participants in the ATL AA cohort. For this analysis, data collected at the 24-30 week visit were assigned as the PUFA consumption values to better reflect dietary patterns during the gestational period when fetal growth is more variable.[25,26] We did not observe a significant difference in PUFA intake values among those who had data available from visit 1 and visit 2 for the eleven PUFAs that consumption was estimated for (Table S9). If FFQ data were available from the first visit but not the second, then these data were used in place (n=262, 45.3%). Participants' dietary data were excluded if analysis revealed an implausible mean caloric intake of <500 or ≥5000 kcal/day

(n=40, 6.9%).[27] While it is necessary to address information bias resulting from completion of the FFQ, we recognize this method may introduce selection bias since exclusion may be associated with maternal weight which is also positively associated with fetal growth.[28]

Using FFQ data, we estimated intake of linoleic acid (LA; 18:2n-6), α-linolenic acid (ALA; 18:3n-3), stearidonic acid (SA; 18:4n-3), arachidonic acid (AA; 20:4n-6), eicosapentaenoic acid (EPA; 20:5n-3), docosapentaenoic acid (DPA; 22:5n-3), docosahexaenoic acid (DHA; 22:6n-3), total n-6, total n-3, total monounsaturated fatty acid (MUFA), and total PUFA intake continuously as grams/day. The MUFA:PUFA and n-6:n-3 ratios were calculated *post hoc*. After inspecting model partial residual plots, FFQ values save the calculated ratios were $\log_2$-transformed to account for a potential log-linear relationship between fatty acid consumption and BWZ.[29] Additionally, participants with values more extreme than ±1.5 time the interquartile range (IQR) were excluded (n=4) to address information bias introduced from inaccurate estimates of nutrient intakes from meal portion sizes and components.[30]

Table S1. Comparison of the distribution of characteristics among study participants with complete birth outcome data and those included in the analytical sample. Continuous variables are reported as mean (standard deviation), and categorical variables are reported as n (%). On average, sex-specific birth weight for gestational age z-score was lower in the analytical sample as was the prevalence of small for gestational age and term low birth weight.

Characteristic	Complete Birth Outcome (N = 521)	Analytical Sample (N = 297)
Daily Caloric Intake (kcal)	-	1,977 (1,005)
Body Mass Index (kg/m^2)	29 (8)	28 (7)
Age (years)	25 (5)	25 (5)
Educational Attainment		
College Graduate or More	82 (16%)	54 (18%)
Some College	201 (39%)	85 (29%)
High School	152 (29%)	111 (37%)
Less Than High School	86 (17%)	47 (16%)
Conception Season		
Winter	87 (17%)	51 (17%)
Spring	116 (22%)	63 (21%)
Summer	164 (31%)	94 (32%)
Fall	154 (30%)	89 (30%)
BWZ[1]	-0.47 (1.05)	-0.55 (1.01)
SGA[2]	116 (22%)	76 (26%)
Term LBW[3]	25 (4.8%)	23 (7.7%)
Supplemental n-3 PUFA	-	14 (4.7%)
Supplemental n-6 PUFA	-	14 (4.7%)

[1] Sex-specific birth weight for gestational age z-score
[2] Small for gestational age
[3] Term low birth weight

Table S2. Comparison of geometric mean (SD) PUFA (grams) from participants who had nutritional assessment data from both visit 1 (n=174) and visit 2 (n=123). Means were not calculated for ratio values. There was not a difference in the mean daily consumption or calculated ratio for each PUFA measure used in this analysis.

PUFA	Visit 1	Visit 2	*p*
LA	12.5 (1.79)	11.64 (1.76)	0.29
ALA	1.29 (1.78)	1.25 (1.73)	0.65
SA	0 (3.9)	0 (4.62)	0.72
AA	0.09 (2.09)	0.1 (2.02)	0.43
EPA	0.01 (3.41)	0.02 (3.69)	0.26
DPA	0 (4.34)	0.01 (3.63)	0.19
DHA	0.03 (2.79)	0.04 (3.04)	0.15
n-6	12.61 (1.79)	11.75 (1.76)	0.3
n-3	1.37 (1.77)	1.36 (1.71)	0.89
MUFA	27.67 (1.76)	25.9 (1.69)	0.3
PUFA	14.91 (1.77)	14.06 (1.75)	0.38
MUFA:PUFA	-	-	-
n-6:n-3	-	-	-

<u>PUFAs:</u>
linoleic acid (LA; 18:2n-6), a-linolenic acid (ALA; 18:3n-3), stearidonic acid (SA; 18:4n-3), arachidonic acid (AA; 20:4n-6), eicosapentaenoic acid (EPA; 20:5n-3), docosapentaenoic acid (DPA; 22:5n-3), docosahexaenoic acid (DHA; 22:6n-3), total n-6 polyunsaturated fatty acid (Total n-6), total n-3 polyunsaturated fatty acid (Total n-3), total monounsaturated fatty acid (MUFA) to total polyunsaturated fatty acid (PUFA) ratio, total n-6 polyunsaturated fatty acid to total n-3 polyunsaturated fatty acid ratio (n-6:n-3)

Table S3. Correlation of average daily traffic-related air pollution (TRAP) exposure during the first trimester (T1) or the full gestational period (Full) for the 297 study participants with complete birth outcome, air pollution exposure assessment, and dietary assessment data. Exposures were estimated by geocoding participants' residential addresses collected during the first prenatal visit (8-14 weeks of gestation) and linking this to daily ambient TRAP concentrations.

	CO (T1)	CO (Full)	NO_x (T1)	NO_x (Full)	$PM_{2.5}$ (T1)	$PM_{2.5}$ (Full)
CO (T1)	1	0.98	0.9	0.95	0.33	0.52
CO (Full)	0.98	1	0.84	0.95	0.37	0.55
NO_x (T1)	0.9	0.84	1	0.91	0.26	0.5
NO_x (Full)	0.95	0.95	0.91	1	0.38	0.58
$PM_{2.5}$ (T1)	0.33	0.37	0.26	0.38	1	0.67
$PM_{2.5}$ (Full)	0.52	0.55	0.5	0.58	0.67	1

TRAPs:
carbon monoxide (CO), nitrogen oxides (NO_x), fine particulate matter ($PM_{2.5}$)

Table S4. Correlation of PUFA consumption among the 297 study participants with complete birth outcome, air pollution exposure assessment, and dietary assessment data. Values were estimated using a modified Block-Bodnar semi-quantitative food frequency questionnaire administered to participants at two prenatal care visits between 8-14 and 24-30 weeks of gestation to assess dietary intake and supplementation over the previous three months.

	LA	ALA	SA	AA	EPA	DPA	DHA	MUFA	PUFA	Total n-6	Total n-3	MUFA: PUFA	n-6: n-3
LA	1	0.95	0.28	0.65	0.32	0.34	0.39	1	0.95	0.95	0.99	-0.68	0.1
ALA	0.95	1	0.25	0.59	0.31	0.33	0.35	0.95	0.99	0.88	0.95	-0.7	0.09
SA	0.28	0.25	1	0.34	0.79	0.66	0.7	0.28	0.34	0.31	0.26	-0.06	0.05
AA	0.65	0.59	0.34	1	0.47	0.53	0.68	0.66	0.62	0.75	0.66	-0.22	0.07
EPA	0.32	0.31	0.79	0.47	1	0.77	0.91	0.32	0.41	0.31	0.32	-0.22	0.1
DPA	0.34	0.33	0.66	0.53	0.77	1	0.75	0.34	0.41	0.35	0.34	-0.18	0.01
DHA	0.39	0.35	0.7	0.68	0.91	0.75	1	0.39	0.45	0.41	0.39	-0.19	0.09
MUFA	1	0.95	0.28	0.66	0.32	0.34	0.39	1	0.95	0.95	0.99	-0.68	0.1
PUFA	0.95	0.99	0.34	0.62	0.41	0.41	0.45	0.95	1	0.88	0.95	-0.69	0.09
Total n-6	0.95	0.88	0.31	0.75	0.31	0.35	0.41	0.95	0.88	1	0.94	-0.45	0.09
Total n-3	0.99	0.95	0.26	0.66	0.32	0.34	0.39	0.99	0.95	0.94	1	-0.7	0.1
MUFA:PUFA	-0.68	-0.7	-0.06	-0.22	-0.22	-0.18	-0.19	-0.68	-0.69	-0.45	-0.7	1	-0.07
n-6:n-3	0.1	0.09	0.05	0.07	0.1	0.01	0.09	0.1	0.09	0.09	0.1	-0.07	1

<u>PUFAs:</u>
linoleic acid (LA; 18:2n-6), a-linolenic acid (ALA; 18:3n-3), stearidonic acid (SA; 18:4n-3), arachidonic acid (AA; 20:4n-6), eicosapentaenoic acid (EPA; 20:5n-3), docosapentaenoic acid (DPA; 22:5n-3), docosahexaenoic acid (DHA; 22:6n-3), total n-6 polyunsaturated fatty acid (Total n-6), total n-3 polyunsaturated fatty acid (Total n-3), total monounsaturated fatty

acid (MUFA) to total polyunsaturated fatty acid (PUFA) ratio, total n-6 polyunsaturated fatty acid to total n-3 polyunsaturated fatty acid ratio (n-6:n-3)

Table S5. Joint mixture associations of a doubling of individual dietary polyunsaturated fatty acid (PUFA) intake during early pregnancy and interquartile range (IQR) change of the average daily traffic-related air pollution (TRAP) during the full gestational period on birth weight z-score (BWZ) adjusted for average daily caloric intake, body mass index at recruitment, age at recruitment, educational attainment, and conception season.

		PUFA	TRAP	PUFA*TRAP	Joint Mixture	*p*-int	*q*-int
LA	CO	**0.6 (0.17, 1.03)***	**-0.05 (-0.11, 0)***	-0.03 (-0.1, 0.04)	**0.51 (0.13, 0.9)***	0.4	0.42
	NO_x	**0.63 (0.23, 1.04)***	-0.05 (-0.1, 0.01)	-0.04 (-0.11, 0.03)	**0.55 (0.2, 0.89)***	0.27	0.34
	$PM_{2.5}$	**1.63 (0.14, 3.12)***	**-0.09 (-0.17, -0.01)***	-0.07 (-0.16, 0.02)	**1.47 (0.11, 2.83)***	0.11	0.31
ALA	CO	**0.5 (0.06, 0.93)***	-0.05 (-0.1, 0)	-0.04 (-0.12, 0.03)	**0.4 (0, 0.8)***	0.26	0.34
	NO_x	**0.55 (0.14, 0.96)***	-0.04 (-0.09, 0.01)	-0.06 (-0.14, 0.02)	**0.45 (0.09, 0.81)***	0.13	0.34
	$PM_{2.5}$	**2.18 (0.61, 3.76)***	**-0.09 (-0.17, -0.01)***	**-0.12 (-0.21, -0.02)***	**1.98 (0.48, 3.47)***	**0.02^**	**0.26**
SA	CO	**0.16 (0.01, 0.31)***	-0.05 (-0.1, 0.01)	**-0.03 (-0.06, 0)***	0.08 (-0.06, 0.23)	**0.04^**	**0.31**
	NO_x	**0.13 (0, 0.27)***	-0.04 (-0.09, 0.01)	-0.03 (-0.06, 0)	0.07 (-0.06, 0.2)	**0.06^**	**0.31**
	$PM_{2.5}$	0.49 (-0.13, 1.12)	**-0.08 (-0.16, 0)***	-0.03 (-0.07, 0.01)	0.38 (-0.14, 0.9)	0.14	0.34
AA	CO	0.17 (-0.11, 0.46)	**-0.06 (-0.11, 0)***	-0.03 (-0.08, 0.02)	0.09 (-0.15, 0.33)	0.26	0.34
	NO_x	0.17 (-0.1, 0.44)	-0.05 (-0.1, 0.01)	-0.03 (-0.09, 0.02)	0.09 (-0.12, 0.31)	0.26	0.34
	$PM_{2.5}$	0.79 (-0.3, 1.88)	**-0.09 (-0.17, -0.01)***	-0.05 (-0.11, 0.02)	0.65 (-0.38, 1.69)	0.17	0.34
EPA	CO	**0.18 (0.02, 0.34)***	-0.05 (-0.1, 0)	-0.03 (-0.05, 0)	0.11 (-0.04, 0.25)	**0.09^**	**0.31**
	NO_x	**0.17 (0.03, 0.32)***	-0.04 (-0.09, 0.01)	-0.03 (-0.06, 0)	0.11 (-0.02, 0.23)	**0.09^**	**0.31**
	$PM_{2.5}$	0.47 (-0.18, 1.11)	**-0.08 (-0.16, 0)***	-0.03 (-0.06, 0.01)	0.36 (-0.21, 0.92)	0.21	0.34
DPA	CO	0.1 (-0.03, 0.23)	**-0.05 (-0.11, 0)***	-0.02 (-0.04, 0)	0.03 (-0.13, 0.18)	0.11	0.31
	NO_x	0.1 (-0.02, 0.22)	-0.04 (-0.09, 0.01)	-0.02 (-0.04, 0)	0.04 (-0.1, 0.18)	**0.09^**	**0.31**
	$PM_{2.5}$	0.45 (-0.22, 1.11)	**-0.08 (-0.16, 0)***	-0.03 (-0.07, 0.01)	0.34 (-0.4, 1.07)	0.19	0.34
DHA	CO	0.17 (-0.02, 0.35)	**-0.05 (-0.11, 0)***	-0.02 (-0.06, 0.01)	0.09 (-0.07, 0.25)	0.15	0.34
	NO_x	0.16 (-0.02, 0.33)	-0.04 (-0.1, 0.01)	-0.02 (-0.06, 0.01)	0.09 (-0.05, 0.23)	0.18	0.34
	$PM_{2.5}$	0.49 (-0.25, 1.23)	**-0.09 (-0.17, -0.01)***	-0.03 (-0.07, 0.02)	0.38 (-0.28, 1.04)	0.23	0.34

**p* <0.05
^*p*-int<0.1
#*q*-int<0.2

PUFAs:
linoleic acid (LA; 18:2n-6), a-linolenic acid (ALA; 18:3n-3), stearidonic acid (SA; 18:4n-3), arachidonic acid (AA; 20:4n-6), eicosapentaenoic acid (EPA; 20:5n-3), docosapentaenoic acid (DPA; 22:5n-3), docosahexaenoic acid (DHA; 22:6n-3)

TRAPs:
carbon monoxide (CO), nitrogen oxides (NO_x), fine particulate matter ($PM_{2.5}$)

Table S6. Joint mixture associations of a doubling of categorized dietary polyunsaturated fatty acid (PUFA) intake during early pregnancy and interquartile range (IQR) change of the average daily traffic-related air pollution (TRAP) during the full gestational period on birth weight z-score (BWZ) adjusted for average daily caloric intake, body mass index at recruitment, age at recruitment, educational attainment, and conception season. PUFAs were log_2-transformed except for ratio values.

		PUFA	TRAP	PUFA*TRAP	Joint Mixture	*p*-int	*q*-int
Total n-6	CO	**0.6 (0.17, 1.03)***	**-0.05 (-0.11, 0)***	-0.03 (-0.1, 0.04)	**0.51 (0.12, 0.9)***	0.4	0.42
	NO_x	**0.63 (0.22, 1.04)***	-0.05 (-0.1, 0.01)	-0.04 (-0.11, 0.03)	**0.55 (0.2, 0.89)***	0.27	0.34
	$PM_{2.5}$	**1.62 (0.13, 3.12)***	**-0.09 (-0.17, -0.01)***	-0.07 (-0.16, 0.02)	**1.46 (0.1, 2.82)***	0.11	0.31
Total n-3	CO	**0.52 (0.09, 0.95)***	-0.05 (-0.1, 0)	-0.05 (-0.12, 0.03)	**0.42 (0.03, 0.82)***	0.21	0.34
	NO_x	**0.57 (0.16, 0.98)***	-0.04 (-0.09, 0.01)	-0.06 (-0.14, 0.02)	**0.47 (0.11, 0.82)***	0.11	0.31
	$PM_{2.5}$	**2.21 (0.63, 3.79)***	**-0.09 (-0.17, -0.01)***	**-0.12 (-0.21, -0.02)***	**2.01 (0.52, 3.49)***	**0.02^**	0.26
MUFA	CO	**0.47 (0.01, 0.94)***	-0.05 (-0.1, 0)	-0.04 (-0.11, 0.04)	0.39 (-0.05, 0.83)	0.33	0.38
	NO_x	**0.5 (0.06, 0.94)***	-0.04 (-0.09, 0.01)	-0.05 (-0.12, 0.03)	**0.41 (0.01, 0.81)***	0.24	0.34
	$PM_{2.5}$	1.15 (-0.41, 2.71)	**-0.08 (-0.16, 0)***	-0.05 (-0.15, 0.04)	1.01 (-0.49, 2.51)	0.27	0.34
PUFA	CO	**0.64 (0.2, 1.08)***	-0.05 (-0.1, 0)	-0.04 (-0.11, 0.03)	**0.55 (0.16, 0.94)***	0.29	0.35
	NO_x	**0.67 (0.25, 1.09)***	-0.04 (-0.09, 0.01)	-0.05 (-0.12, 0.02)	**0.58 (0.24, 0.93)***	0.18	0.34
	$PM_{2.5}$	**1.79 (0.27, 3.3)***	**-0.09 (-0.16, -0.01)***	-0.08 (-0.17, 0.01)	**1.62 (0.23, 3.02)***	**0.08^**	0.31
MUFA:PUFA	CO	-0.35 (-1.26, 0.57)	-0.07 (-0.41, 0.27)	0.01 (-0.17, 0.19)	-0.41 (-1.37, 0.55)	0.92	0.92
	NO_x	-0.51 (-1.29, 0.28)	-0.13 (-0.44, 0.18)	0.05 (-0.12, 0.21)	-0.59 (-1.36, 0.18)	0.58	0.59
	$PM_{2.5}$	-4.12 (-8.34, 0.09)	**-0.53 (-1.03, -0.04)***	0.24 (-0.02, 0.5)	**-4.42 (-8.47, -0.37)***	**0.07^**	0.31
n-6:n-3	CO	-0.02 (-0.15, 0.12)	-0.17 (-0.41, 0.06)	0.01 (-0.01, 0.03)	-0.18 (-0.43, 0.07)	0.31	0.37
	NO_x	-0.01 (-0.13, 0.12)	-0.15 (-0.39, 0.09)	0.01 (-0.01, 0.03)	-0.14 (-0.39, 0.1)	0.39	0.42

	$PM_{2.5}$	**-0.72 (-1.36, -0.09)***	**-0.53 (-0.91, -0.16)***	**0.05 (0.01, 0.09)***	**-1.21 (-2, -0.42)***	**0.02^**	0.26

*p <0.05
^p-int<0.1
#q-int<0.2

PUFAs:
total n-6 polyunsaturated fatty acid (Total n-6), total n-3 polyunsaturated fatty acid (Total n-3), total monounsaturated fatty acid (MUFA) to total polyunsaturated fatty acid (PUFA) ratio, total n-6 polyunsaturated fatty acid to total n-3 polyunsaturated fatty acid ratio (n-6:n-3)

TRAPs:
carbon monoxide (CO), nitrogen oxides (NO_x), fine particulate matter ($PM_{2.5}$)

Table S7. Prevalence ratios and 95% confidence intervals for the joint mixture associations of a doubling of individual dietary polyunsaturated fatty acid (PUFA) intake during early pregnancy and interquartile range (IQR) change of the average daily traffic-related air pollution (TRAP) during the first trimester on small for gestational age adjusted for average daily caloric intake, body mass index at recruitment, age at recruitment, educational attainment, and conception season.

		PUFA	TRAP	PUFA*TRAP	Joint Mixture	*p*-int	*q*-int
LA	CO	**0.49 (0.27, 0.9)***	**1.07 (1, 1.14)***	1.02 (0.94, 1.11)	**0.53 (0.3, 0.93)***	0.7	0.80
	NO_x	**0.48 (0.25, 0.91)***	**1.09 (1.01, 1.18)***	1.03 (0.93, 1.15)	**0.54 (0.31, 0.95)***	0.66	0.78
	$PM_{2.5}$	**0.19 (0.04, 0.79)***	**1.18 (1, 1.39)***	1.14 (0.98, 1.32)	**0.25 (0.07, 0.9)***	0.2	0.37
ALA	CO	0.54 (0.28, 1.03)	1.06 (0.99, 1.13)	1.04 (0.94, 1.14)	0.59 (0.33, 1.06)	0.56	0.75
	NO_x	0.55 (0.28, 1.09)	1.08 (1, 1.17)	1.04 (0.92, 1.17)	0.62 (0.34, 1.12)	0.64	0.78
	$PM_{2.5}$	**0.18 (0.05, 0.71)***	**1.19 (1.01, 1.4)***	1.16 (1, 1.34)	**0.25 (0.07, 0.83)***	0.14	0.31
SA	CO	**0.78 (0.64, 0.93)***	1.02 (0.93, 1.11)	**1.05 (1.02, 1.09)***	0.83 (0.67, 1.04)	**0.04^**	**0.14#**
	NO_x	**0.77 (0.64, 0.92)***	1.04 (0.93, 1.16)	**1.07 (1.02, 1.13)***	0.86 (0.69, 1.07)	**0.03^**	**0.12#**
	$PM_{2.5}$	0.84 (0.52, 1.36)	**1.18 (1, 1.4)***	1.02 (0.96, 1.08)	1.01 (0.64, 1.61)	0.65	0.78
AA	CO	0.79 (0.53, 1.18)	1.06 (0.99, 1.13)	1.03 (0.97, 1.09)	0.86 (0.59, 1.24)	0.54	0.74
	NO_x	0.82 (0.55, 1.21)	1.08 (0.99, 1.17)	1.03 (0.95, 1.11)	0.9 (0.64, 1.28)	0.62	0.78
	$PM_{2.5}$	**0.29 (0.1, 0.86)***	**1.19 (1.01, 1.4)***	**1.14 (1, 1.29)***	0.39 (0.15, 1.04)	0.11	0.26
EPA	CO	**0.75 (0.6, 0.94)***	1.04 (0.96, 1.13)	**1.04 (1, 1.08)***	0.81 (0.63, 1.03)	0.1	0.26
	NO_x	**0.77 (0.63, 0.94)***	1.07 (0.98, 1.17)	**1.05 (1.01, 1.09)***	0.86 (0.69, 1.06)	0.11	0.26
	$PM_{2.5}$	0.66 (0.35, 1.25)	**1.19 (1.01, 1.4)***	1.04 (0.97, 1.11)	0.82 (0.45, 1.47)	0.45	0.69
DPA	CO	**0.81 (0.69, 0.96)***	1.05 (0.97, 1.13)	**1.03 (1, 1.07)***	0.88 (0.73, 1.07)	0.11	0.26
	NO_x	**0.8 (0.69, 0.94)***	1.07 (0.97, 1.17)	**1.05 (1.01, 1.09)***	0.9 (0.75, 1.07)	**0.07^**	0.23
	$PM_{2.5}$	0.86 (0.46, 1.63)	**1.19 (1.01, 1.4)***	1.01 (0.94, 1.09)	1.04 (0.57, 1.89)	0.8	0.85
DHA	CO	0.79 (0.62, 1.01)	1.05 (0.98, 1.13)	1.03 (1, 1.07)	0.86 (0.67, 1.11)	0.22	0.40
	NO_x	0.81 (0.65, 1.02)	1.08 (0.99, 1.17)	1.04 (0.99, 1.08)	0.91 (0.72, 1.14)	0.26	0.45
	$PM_{2.5}$	0.66 (0.32, 1.39)	**1.19 (1, 1.4)***	1.04 (0.96, 1.13)	0.82 (0.41, 1.62)	0.49	0.72

*p <0.05
^p-int<0.1
#q-int<0.2

PUFAs:
linoleic acid (LA; 18:2n-6), a-linolenic acid (ALA; 18:3n-3), stearidonic acid (SA; 18:4n-3), arachidonic acid (AA; 20:4n-6), eicosapentaenoic acid (EPA; 20:5n-3), docosapentaenoic acid (DPA; 22:5n-3), docosahexaenoic acid (DHA; 22:6n-3)

TRAPs:
carbon monoxide (CO), nitrogen oxides (NO_x), fine particulate matter ($PM_{2.5}$)

Table S8. Prevalence ratios and 95% confidence intervals for the joint mixture associations of a doubling of categorized dietary polyunsaturated fatty acid (PUFA) intake during early pregnancy and interquartile range (IQR) change of the average daily traffic-related air pollution (TRAP) during the first trimester on small for gestational age adjusted for average daily caloric intake, body mass index at recruitment, age at recruitment, educational attainment, and conception season. PUFAs were log_2-transformed except for ratio values.

		PUFA	TRAP	PUFA*TRAP	Joint Mixture	*p*-int	*q*-int
Total n-6	CO	**0.49 (0.27, 0.9)***	**1.07 (1, 1.14)***	1.02 (0.94, 1.11)	**0.53 (0.3, 0.93)***	0.69	0.80
	NO_x	**0.48 (0.25, 0.91)***	**1.09 (1.01, 1.18)***	1.03 (0.93, 1.15)	**0.54 (0.31, 0.95)***	0.66	0.78
	$PM_{2.5}$	**0.18 (0.04, 0.79)***	**1.18 (1, 1.39)***	1.14 (0.98, 1.33)	**0.25 (0.07, 0.9)***	0.2	0.37
Total n-3	CO	**0.52 (0.27, 0.98)***	1.05 (0.99, 1.13)	1.05 (0.96, 1.15)	0.57 (0.32, 1.03)	0.45	0.69
	NO_x	0.53 (0.27, 1.02)	1.08 (1, 1.17)	1.05 (0.94, 1.18)	0.6 (0.34, 1.08)	0.5	0.72
	$PM_{2.5}$	**0.19 (0.05, 0.77)***	**1.19 (1.01, 1.4)***	1.15 (0.99, 1.34)	**0.26 (0.08, 0.9)***	0.16	0.33
MUFA	CO	**0.5 (0.26, 0.94)***	1.06 (0.99, 1.13)	1.03 (0.94, 1.13)	**0.54 (0.3, 0.98)***	0.6	0.78
	NO_x	**0.49 (0.25, 0.94)***	1.08 (0.99, 1.17)	1.05 (0.94, 1.17)	**0.55 (0.31, 0.98)***	0.52	0.74
	$PM_{2.5}$	**0.17 (0.04, 0.78)***	1.16 (0.99, 1.37)	1.16 (0.98, 1.36)	**0.23 (0.06, 0.89)***	0.17	0.34
PUFA	CO	**0.43 (0.23, 0.79)***	1.06 (1, 1.13)	1.03 (0.95, 1.12)	**0.47 (0.27, 0.83)***	0.57	0.76
	NO_x	**0.41 (0.21, 0.8)***	**1.09 (1, 1.17)***	1.05 (0.94, 1.17)	**0.47 (0.26, 0.84)***	0.5	0.72
	$PM_{2.5}$	**0.16 (0.04, 0.69)***	1.17 (1, 1.38)	1.14 (0.98, 1.33)	**0.22 (0.06, 0.79)***	0.19	0.37
MUFA:PUFA	CO	1.42 (0.37, 5.34)	1.08 (0.7, 1.67)	0.99 (0.78, 1.25)	1.52 (0.34, 6.87)	0.95	0.96
	NO_x	1.27 (0.32, 5.04)	1.04 (0.59, 1.86)	1.02 (0.75, 1.4)	1.35 (0.26, 6.87)	0.91	0.94
	$PM_{2.5}$	3.15 (0.12, 86.05)	1.43 (0.7, 2.9)	0.91 (0.63, 1.31)	4.1 (0.11, 155.44)	0.72	0.80
n-6:n-3	CO	1.08 (0.88, 1.33)	**1.39 (1.04, 1.86)***	0.97 (0.94, 1)	1.46 (0.93, 2.29)	0.26	0.45
	NO_x	1.06 (0.86, 1.31)	1.46 (0.94, 2.24)	0.97 (0.92, 1.02)	1.49 (0.84, 2.66)	0.35	0.59
	$PM_{2.5}$	1.39 (0.62, 3.09)	1.77 (0.79, 3.95)	0.96 (0.88, 1.05)	2.35 (0.52, 10.59)	0.42	0.67

*p <0.05
^p-int<0.1
#q-int<0.2

PUFAs:
total n-6 polyunsaturated fatty acid (Total n-6), total n-3 polyunsaturated fatty acid (Total n-3), total monounsaturated fatty acid (MUFA) to total polyunsaturated fatty acid (PUFA) ratio, total n-6 polyunsaturated fatty acid to total n-3 polyunsaturated fatty acid ratio (n-6:n-3)

TRAPs:
carbon monoxide (CO), nitrogen oxides (NO_x), fine particulate matter ($PM_{2.5}$)

Table S9. Prevalence ratios and 95% confidence intervals for the joint mixture associations of a doubling of individual dietary polyunsaturated fatty acid (PUFA) intake during early pregnancy and interquartile range (IQR) change of the average daily traffic-related air pollution (TRAP) during the first trimester on term low birth weight adjusted for average daily caloric intake, body mass index at recruitment, age at recruitment, educational attainment, and conception season.

		PUFA	TRAP	PUFA*TRAP	Joint Mixture	*p*-int	*q*-int
LA	CO	**0.11 (0.03, 0.44)***	0.9 (0.73, 1.13)	**1.37 (1.1, 1.7)***	**0.14 (0.04, 0.51)***	**0.01^**	**0.06#**
	NO_x	**0.14 (0.03, 0.54)***	0.96 (0.78, 1.18)	**1.47 (1.11, 1.93)***	**0.19 (0.06, 0.62)***	**0.01^**	**0.06#**
	$PM_{2.5}$	**0.04 (0, 0.91)***	1.03 (0.75, 1.4)	1.36 (0.92, 2.01)	**0.05 (0, 0.84)***	0.1	0.26
ALA	CO	**0.09 (0.01, 0.58)***	0.85 (0.66, 1.1)	**1.68 (1.17, 2.4)***	**0.13 (0.02, 0.71)***	**<0.01^**	**<0.01#**
	NO_x	**0.17 (0.04, 0.81)***	0.93 (0.75, 1.15)	**1.6 (1.15, 2.22)***	**0.25 (0.07, 0.96)***	**0.01^**	**0.06#**
	$PM_{2.5}$	0.06 (0, 1.5)	1.02 (0.74, 1.39)	1.36 (0.93, 1.99)	0.08 (0, 1.44)	0.11	0.26
SA	CO	0.67 (0.41, 1.07)	0.88 (0.68, 1.14)	**1.13 (1.02, 1.25)***	0.66 (0.37, 1.19)	**0.03^**	**0.12#**
	NO_x	0.71 (0.47, 1.05)	0.9 (0.69, 1.16)	**1.15 (1.04, 1.28)***	0.73 (0.45, 1.18)	**0.04^**	**0.14#**
	$PM_{2.5}$	0.92 (0.36, 2.32)	0.99 (0.73, 1.34)	1.03 (0.92, 1.14)	0.93 (0.4, 2.16)	0.77	0.83
AA	CO	**0.24 (0.09, 0.62)***	0.85 (0.65, 1.11)	**1.39 (1.13, 1.72)***	**0.28 (0.11, 0.74)***	**<0.01^**	**<0.01#**
	NO_x	**0.4 (0.21, 0.75)***	0.94 (0.74, 1.2)	**1.34 (1.15, 1.57)***	**0.5 (0.28, 0.91)***	**0.01^**	**0.06#**
	$PM_{2.5}$	0.29 (0.02, 3.59)	1.01 (0.73, 1.38)	1.15 (0.87, 1.53)	0.34 (0.04, 3.16)	0.37	0.61
EPA	CO	0.7 (0.43, 1.16)	0.91 (0.71, 1.17)	**1.11 (1, 1.22)***	0.71 (0.39, 1.3)	**0.06^**	0.20
	NO_x	0.77 (0.49, 1.2)	0.94 (0.71, 1.24)	1.12 (1, 1.25)	0.8 (0.48, 1.36)	**0.08^**	0.25
	$PM_{2.5}$	0.77 (0.25, 2.35)	1 (0.73, 1.38)	1.05 (0.93, 1.18)	0.81 (0.28, 2.32)	0.63	0.78
DPA	CO	0.59 (0.35, 1.01)	0.84 (0.61, 1.16)	**1.16 (1.02, 1.31)***	0.58 (0.29, 1.16)	**0.02^**	**0.09#**
	NO_x	0.7 (0.48, 1.01)	0.87 (0.64, 1.18)	**1.17 (1.05, 1.3)***	0.71 (0.44, 1.16)	**0.02^**	**0.09#**
	$PM_{2.5}$	0.52 (0.12, 2.26)	0.98 (0.71, 1.35)	1.09 (0.92, 1.3)	0.56 (0.14, 2.25)	0.41	0.67
DHA	CO	0.56 (0.3, 1.07)	0.88 (0.63, 1.23)	**1.16 (1, 1.34)***	0.57 (0.26, 1.27)	**0.02^**	**0.09#**
	NO_x	0.68 (0.43, 1.07)	0.93 (0.68, 1.28)	**1.16 (1.02, 1.31)***	0.73 (0.42, 1.28)	**0.04^**	**0.14#**
	$PM_{2.5}$	1.19 (0.26, 5.42)	1.02 (0.74, 1.4)	1 (0.84, 1.18)	1.2 (0.3, 4.8)	0.97	0.97

*p <0.05
^p-int<0.1
#q-int<0.2

PUFAs:
linoleic acid (LA; 18:2n-6), a-linolenic acid (ALA; 18:3n-3), stearidonic acid (SA; 18:4n-3), arachidonic acid (AA; 20:4n-6), eicosapentaenoic acid (EPA; 20:5n-3), docosapentaenoic acid (DPA; 22:5n-3), docosahexaenoic acid (DHA; 22:6n-3)

TRAPs:
carbon monoxide (CO), nitrogen oxides (NO_x), fine particulate matter ($PM_{2.5}$)

Table S10. Prevalence ratios and 95% confidence intervals for the joint mixture associations of a doubling of categorized dietary polyunsaturated fatty acid (PUFA) intake during early pregnancy and interquartile range (IQR) change of the average daily traffic-related air pollution (TRAP) during the first trimester on term low birth weight adjusted for average daily caloric intake, body mass index at recruitment, age at recruitment, educational attainment, and conception season. PUFAs were log_2-transformed except for ratio values.

		PUFA	TRAP	PUFA*TRAP	Joint Mixture	*p*-int	*q*-int
Total n-6	CO	**0.11 (0.03, 0.44)***	0.9 (0.72, 1.12)	**1.37 (1.1, 1.71)***	**0.14 (0.04, 0.51)***	**0.01^**	**0.06#**
	NO_x	**0.14 (0.03, 0.53)***	0.96 (0.78, 1.18)	**1.47 (1.12, 1.94)***	**0.19 (0.06, 0.62)***	**0.01^**	**0.06#**
	$PM_{2.5}$	**0.04 (0, 0.92)***	1.02 (0.75, 1.4)	1.36 (0.92, 2.01)	**0.05 (0, 0.85)***	0.1	0.26
Total n-3	CO	**0.09 (0.02, 0.53)***	0.83 (0.64, 1.08)	**1.71 (1.22, 2.39)***	**0.13 (0.02, 0.66)***	**<0.01^**	**<0.01#**
	NO_x	**0.17 (0.04, 0.76)***	0.91 (0.73, 1.14)	**1.62 (1.2, 2.17)***	**0.25 (0.07, 0.93)***	**<0.01^**	**<0.01#**
	$PM_{2.5}$	0.06 (0, 1.85)	1.02 (0.74, 1.39)	1.35 (0.9, 2.02)	0.09 (0, 1.73)	0.13	0.30
MUFA	CO	**0.1 (0.02, 0.46)***	0.88 (0.71, 1.09)	**1.49 (1.14, 1.96)***	**0.13 (0.03, 0.55)***	**<0.01^**	**<0.01#**
	NO_x	**0.13 (0.03, 0.55)***	0.94 (0.77, 1.14)	**1.59 (1.18, 2.13)***	**0.2 (0.06, 0.69)***	**<0.01^**	**<0.01#**
	$PM_{2.5}$	0.05 (0, 1.26)	1 (0.73, 1.37)	1.32 (0.91, 1.91)	0.06 (0, 1.13)	0.15	0.33
PUFA	CO	**0.09 (0.02, 0.4)***	0.9 (0.73, 1.12)	**1.39 (1.11, 1.73)***	**0.11 (0.03, 0.47)***	**0.01^**	**0.06#**
	NO_x	**0.11 (0.02, 0.48)***	0.96 (0.78, 1.18)	**1.5 (1.13, 2)***	**0.15 (0.04, 0.56)***	**0.01^**	**0.06#**
	$PM_{2.5}$	**0.03 (0, 0.8)***	1.02 (0.75, 1.4)	1.37 (0.93, 2.02)	**0.04 (0, 0.74)***	**0.09^**	0.26
MUFA:PUFA	CO	1.75 (0.12, 25.68)	1.09 (0.43, 2.74)	0.97 (0.59, 1.6)	1.85 (0.09, 39.87)	0.92	0.94
	NO_x	0.91 (0.06, 13.57)	0.81 (0.25, 2.63)	1.14 (0.62, 2.09)	0.84 (0.03, 21.09)	0.71	0.80
	$PM_{2.5}$	**464.25 (1.8, 119837.46)***	**3.57 (1.1, 11.59)***	**0.52 (0.29, 0.93)***	**857.13 (1.9, 385670.96)***	0.16	0.33
n-6:n-3	CO	0.77 (0.5, 1.16)	0.98 (0.49, 1.96)	1.01 (0.95, 1.07)	0.75 (0.28, 2.04)	0.88	0.93
	NO_x	0.73 (0.48, 1.11)	0.85 (0.35, 2.08)	1.02 (0.94, 1.12)	0.64 (0.2, 2.02)	0.74	0.81
	$PM_{2.5}$	0.46 (0.12, 1.8)	0.58 (0.17, 1.97)	1.07 (0.92, 1.23)	0.28 (0.02, 3.17)	0.54	0.74

*p <0.05
^p-int<0.1

#*q*-int<0.2

PUFAs:
total n-6 polyunsaturated fatty acid (Total n-6), total n-3 polyunsaturated fatty acid (Total n-3), total monounsaturated fatty acid (MUFA) to total polyunsaturated fatty acid (PUFA) ratio, total n-6 polyunsaturated fatty acid to total n-3 polyunsaturated fatty acid ratio (n-6:n-3)

TRAPs:
carbon monoxide (CO), nitrogen oxides (NO_x), fine particulate matter ($PM_{2.5}$)

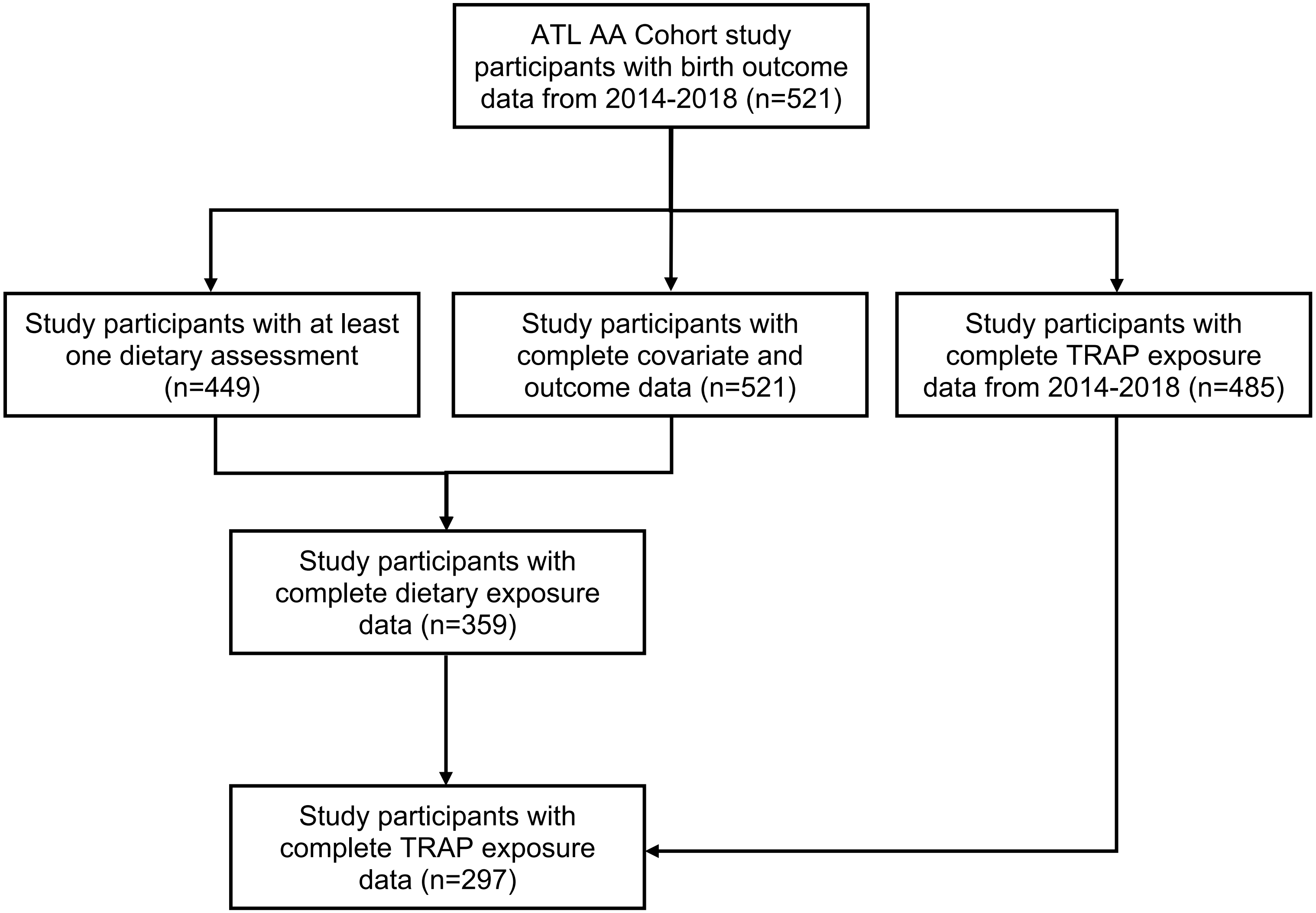

Figure S1. Flow chart depicting the inclusion of 297 study participants from the Atlanta African American Maternal-Child Cohort (ATL AA Cohort) in the present analysis.

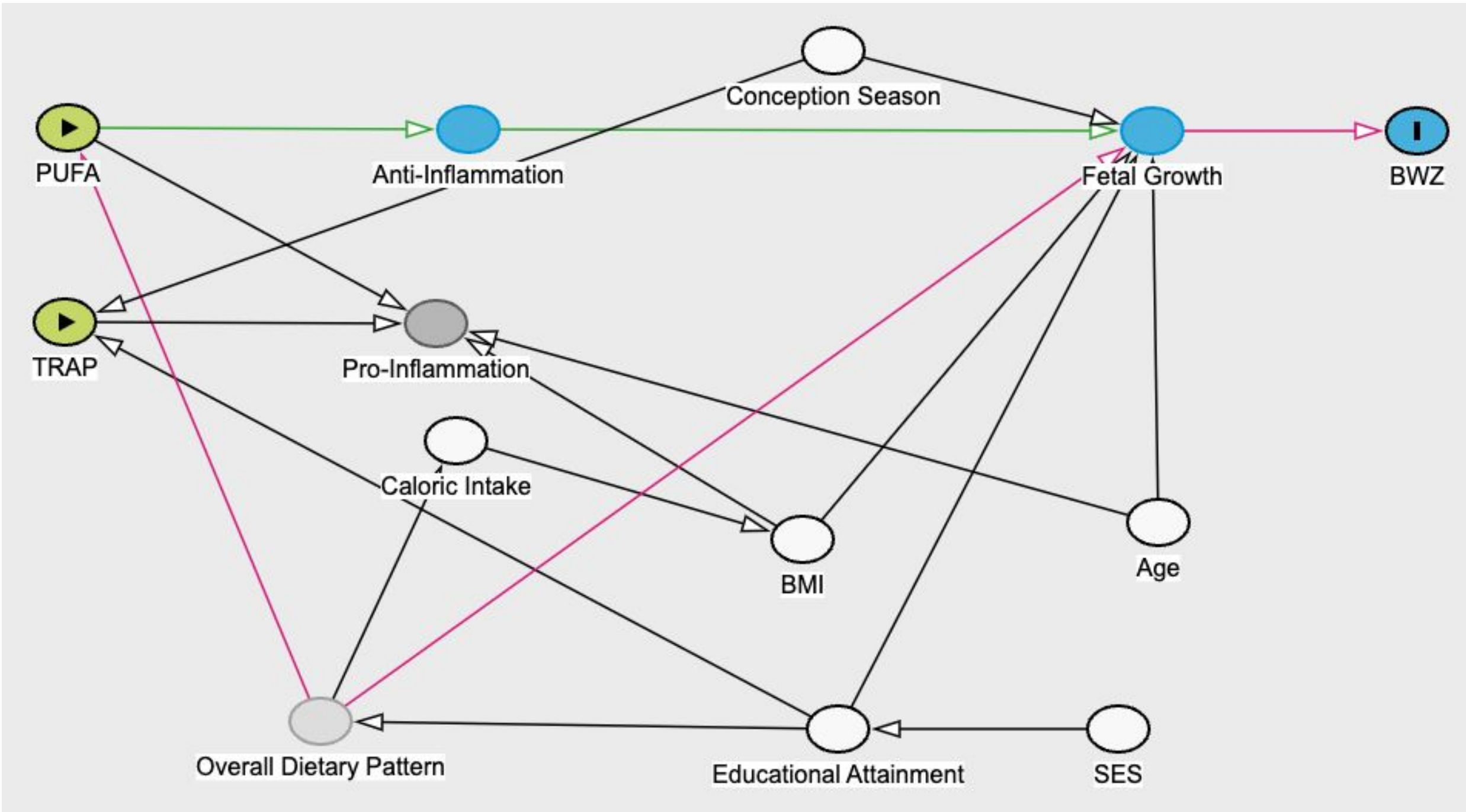

Figure S2. Directed acyclic graph depicting the theorized relationships among dietary polyunsaturated fatty acid (PUFA) intake, traffic-related air pollution (TRAP) exposure, caloric intake, body mass index (BMI), educational attainment, age, conception season, and birth weight z-score (BWZ). Based on the weather pattern in Atlanta, winter was defined as the period between December 22-March 19, spring was defined as the period between March 20-June 20, summer was defined as the period between June 21-September 22, and fall was defined as the period between September 23-December 21.
